# Supplementary material for: Connectivity in eQTL networks dictates reproducibility and genomic properties
Source: Cell Rep Methods. 2022 May 23;2(5):100218. doi: 10.1016/j.crmeth.2022.100218 (PMC9142682; doi:10.1016/j.crmeth.2022.100218)
Supplement: Document S1. Tables S1–S12 [file mmc1.pdf]

Cell Reports Methods, Volume *2*

Supplemental information

Connectivity in eQTL networks dictates reproducibility and genomic properties

Sheila M. Gaynor, Maud Fagny, Xihong Lin, John Platig, and John Quackenbush

Table S 1. eQTL findings using the GTEx Version 8.0 whole genome sequencing and RNA-seq data from the present analysis using MatrixEQTL and the GTEx consortium results. Related to STAR methods and Figure 1

Tissue	Samples	*cis*-eQTLs (Present)	*trans*-eQTLs (Present)	*cis*-eQTLs (GTEx)	*cis*-eQTLs (Shared)	Proportion
Adipose - subcutaneous	581	3153343	82228	2930370	2247923	76.7%
Adipose - visceral	469	2305141	62817	2031091	1592478	78.4%
Adrenal gland	233	1182630	23445	959376	784325	81.8%
Artery - aorta	387	2337275	44445	2038599	1605742	78.8%
Artery - coronary	213	910941	19428	721637	593406	82.2%
Artery - tibial	584	3180675	72189	2958254	2258434	76.3%
Brain - cerebellum	209	1807794	28546	1505827	1221888	81.1%
Brain - cortex	205	1286185	24635	1062868	866891	81.6%
Brain - nucleus accumbens	202	1108374	21265	908866	746800	82.2%
Cells - cultured fibroblasts	483	3018543	81912	2890486	2195677	76.0%
Colon - sigmoid	318	1747716	33670	1474957	1182458	80.2%
Colon - transverse	368	1925725	47708	1672709	1321602	79.0%
Esophagus - gastroesophageal junction	330	1863862	37980	1556679	1238733	79.6%
Esophagus - mucosa	497	2850186	74432	2616828	2028925	77.5%
Esophagus - muscularis	465	2782602	62711	2529273	1958724	77.4%
Heart - atrial appendage	372	1887335	33613	1620487	1282994	79.2%
Heart - left ventricle	386	1654386	34254	1409424	1121382	79.6%
Liver	208	806182	18037	629559	523509	83.2%
Lung	515	2684713	69789	2392204	1872011	78.3%
Muscle Skeletal	706	2781020	67568	2601575	2004324	77.0%
Nerve - tibial	532	3738663	86377	3460273	2648324	76.5%
Pancreas	305	1605071	29111	1340448	1069491	79.8%
Pituitary	237	1505724	30499	1226883	999098	81.4%
Skin - not sun exposed	517	2898044	80477	2697337	2090964	77.5%
Skin - sun exposed	605	3406583	97946	3244342	2488165	76.7%
Spleen	227	1503880	30258	1262024	1019990	80.8%
Stomach	324	1387641	29666	1156158	929380	80.4%
Thyroid	574	3930834	110952	3712240	2832547	76.3%
Whole blood	670	2660893	83035	2414653	1860924	77.1%

Table S 2. Correlation of weighted and unweighted degree measures for the sparse degrees defined by Q-value (QV), local FDR (LFDR), and Benjamini-Hochberg (BH). The correlation is provided for both SNP and gene degree across all values of τ considered, where the weight in the weighted network is defined by the Z-statistic. Related to STAR methods and Figure 2

	SNP			Gene		
τ	QV	LFDR	BH	QV	LFDR	BH
0.05	0.87 (0.021)	0.864 (0.018)	0.868 (0.015)	0.863 (0.02)	0.866 (0.018)	0.857 (0.017)
0.1	0.866 (0.019)	0.855 (0.016)	0.868 (0.015)	0.86 (0.019)	0.862 (0.017)	0.85 (0.016)
0.2	0.863 (0.016)	0.844 (0.015)	0.867 (0.016)	0.855 (0.018)	0.854 (0.016)	0.836 (0.017)
0.25	0.861 (0.015)	0.84 (0.024)	0.866 (0.023)	0.853 (0.018)	0.85 (0.016)	0.827 (0.018)

Table S 3. Average correlation of degrees within tissues for all, cis, and $trans$ edges. For each tissue, we calculated the SNP and gene degree for all nodes in split-sample networks, calculated the correlation between splits, and averaged across five iterations. We report the average across all tissues. Related to Figure 3

		SNP			Gene		
	τ	QV	LFDR	BH	QV	LFDR	BH
Unweighted all	0.05	-0.057 (0.195)	0.014 (0.209)	0.214 (0.148)	0.497 (0.106)	0.454 (0.124)	0.389 (0.171)
	0.1	-0.104 (0.168)	-0.082 (0.2)	0.176 (0.15)	0.51 (0.095)	0.475 (0.112)	0.402 (0.189)
	0.15	-0.102 (0.144)	-0.126 (0.18)	0.145 (0.153)	0.519 (0.087)	0.479 (0.107)	0.432 (0.177)
	0.2	-0.082 (0.124)	-0.141 (0.159)	0.12 (0.157)	0.525 (0.082)	0.48 (0.103)	0.457 (0.158)
	0.25	-0.056 (0.109)	-0.138 (0.139)	0.104 (0.162)	0.527 (0.079)	0.479 (0.099)	0.475 (0.138)
Weighted all	0.05	0.047 (0.208)	0.18 (0.222)	0.467 (0.118)	0.478 (0.118)	0.432 (0.133)	0.402 (0.172)
	0.1	-0.039 (0.181)	0.037 (0.218)	0.419 (0.121)	0.48 (0.112)	0.447 (0.123)	0.414 (0.192)
	0.15	-0.06 (0.155)	-0.038 (0.199)	0.375 (0.124)	0.479 (0.107)	0.445 (0.119)	0.445 (0.182)
	0.2	-0.056 (0.135)	-0.075 (0.177)	0.336 (0.128)	0.476 (0.104)	0.441 (0.117)	0.471 (0.164)
	0.25	-0.041 (0.117)	-0.089 (0.156)	0.302 (0.134)	0.47 (0.102)	0.436 (0.113)	0.491 (0.146)
Unweighted cis	0.05	0.052 (0.188)	0.127 (0.184)	0.208 (0.149)	0.517 (0.104)	0.432 (0.151)	0.384 (0.172)
	0.1	-0.008 (0.18)	0.06 (0.189)	0.171 (0.151)	0.555 (0.077)	0.497 (0.12)	0.395 (0.189)
	0.15	-0.032 (0.166)	0.014 (0.185)	0.141 (0.154)	0.565 (0.067)	0.534 (0.098)	0.425 (0.178)
	0.2	-0.039 (0.153)	-0.016 (0.176)	0.117 (0.158)	0.568 (0.062)	0.544 (0.088)	0.45 (0.157)
	0.25	-0.036 (0.141)	-0.035 (0.166)	0.102 (0.163)	0.571 (0.058)	0.544 (0.083)	0.467 (0.137)
Weighted cis	0.05	0.279 (0.185)	0.418 (0.157)	0.465 (0.119)	0.526 (0.113)	0.425 (0.163)	0.396 (0.173)
	0.1	0.189 (0.189)	0.341 (0.178)	0.419 (0.122)	0.566 (0.087)	0.492 (0.13)	0.408 (0.192)
	0.15	0.142 (0.181)	0.277 (0.187)	0.376 (0.125)	0.577 (0.079)	0.531 (0.108)	0.439 (0.182)
	0.2	0.118 (0.171)	0.228 (0.188)	0.338 (0.129)	0.582 (0.074)	0.544 (0.098)	0.465 (0.164)
	0.25	0.11 (0.161)	0.192 (0.184)	0.305 (0.135)	0.587 (0.071)	0.546 (0.094)	0.485 (0.145)
Unweighted $trans$	0.05	-0.813 (0.048)	-0.806 (0.083)	0.086 (0.087)	0.023 (0.008)	-0.057 (0.018)	0.059 (0.097)
	0.1	-0.767 (0.025)	-0.822 (0.043)	0.007 (0.094)	0.029 (0.006)	0.027 (0.007)	-0.249 (0.078)
	0.15	-0.709 (0.016)	-0.792 (0.026)	-0.064 (0.094)	0.035 (0.007)	0.033 (0.006)	-0.403 (0.052)
	0.2	-0.651 (0.012)	-0.748 (0.017)	-0.134 (0.091)	0.046 (0.015)	0.041 (0.011)	-0.49 (0.029)
	0.25	-0.595 (0.01)	-0.697 (0.012)	-0.2 (0.087)	0.061 (0.027)	0.054 (0.021)	-0.539 (0.016)
Weighted $trans$	0.05	-0.676 (0.068)	-0.596 (0.12)	0.552 (0.089)	0.023 (0.008)	-0.057 (0.018)	0.082 (0.093)
	0.1	-0.683 (0.036)	-0.678 (0.065)	0.508 (0.09)	0.029 (0.006)	0.027 (0.007)	-0.227 (0.075)
	0.15	-0.653 (0.024)	-0.686 (0.042)	0.456 (0.091)	0.035 (0.007)	0.033 (0.006)	-0.382 (0.05)
	0.2	-0.612 (0.018)	-0.668 (0.029)	0.394 (0.091)	0.044 (0.012)	0.041 (0.009)	-0.469 (0.027)
	0.25	-0.567 (0.014)	-0.638 (0.021)	0.327 (0.088)	0.058 (0.023)	0.052 (0.018)	-0.519 (0.016)

Table S 4. Average correlation of degrees between twenty-nine tissues for all, cis, and $trans$ edges. For each sparse degree measure, we calculated the correlation of the degree between each tissue and averaged across all tissue pairings. Related to Figure 4

		SNP			Gene		
	τ	QV	LFDR	BH	QV	LFDR	BH
Unweighted	0.05	0.11 (0.11)	0.149 (0.115)	0.257 (0.101)	0.369 (0.093)	0.336 (0.1)	0.295 (0.123)
	0.1	0.075 (0.102)	0.093 (0.114)	0.253 (0.097)	0.377 (0.09)	0.356 (0.093)	0.336 (0.11)
	0.15	0.07 (0.093)	0.058 (0.109)	0.251 (0.095)	0.383 (0.088)	0.361 (0.091)	0.355 (0.101)
	0.2	0.077 (0.084)	0.04 (0.102)	0.255 (0.094)	0.388 (0.085)	0.364 (0.089)	0.363 (0.096)
	0.25	0.089 (0.076)	0.032 (0.094)	0.265 (0.094)	0.393 (0.082)	0.367 (0.086)	0.368 (0.093)
Weighted	0.05	0.196 (0.116)	0.261 (0.121)	0.375 (0.107)	0.369 (0.096)	0.334 (0.101)	0.306 (0.125)
	0.1	0.14 (0.108)	0.19 (0.12)	0.367 (0.1)	0.373 (0.093)	0.353 (0.094)	0.348 (0.113)
	0.15	0.119 (0.1)	0.141 (0.116)	0.36 (0.096)	0.376 (0.091)	0.357 (0.092)	0.368 (0.105)
	0.2	0.114 (0.091)	0.111 (0.11)	0.355 (0.093)	0.378 (0.088)	0.358 (0.09)	0.378 (0.1)
	0.25	0.116 (0.083)	0.093 (0.102)	0.356 (0.092)	0.379 (0.085)	0.36 (0.087)	0.385 (0.098)
Unweighted	0.05	0.175 (0.104)	0.208 (0.107)	0.252 (0.101)	0.36 (0.1)	0.285 (0.12)	0.289 (0.123)
cis	0.1	0.147 (0.1)	0.182 (0.105)	0.249 (0.097)	0.377 (0.093)	0.342 (0.106)	0.33 (0.11)
	0.15	0.134 (0.096)	0.16 (0.103)	0.248 (0.095)	0.383 (0.091)	0.364 (0.099)	0.347 (0.101)
	0.2	0.129 (0.092)	0.142 (0.101)	0.253 (0.094)	0.386 (0.089)	0.371 (0.095)	0.354 (0.095)
	0.25	0.13 (0.086)	0.13 (0.097)	0.263 (0.095)	0.388 (0.088)	0.373 (0.093)	0.356 (0.091)
Weighted	0.05	0.318 (0.108)	0.358 (0.112)	0.372 (0.107)	0.371 (0.104)	0.293 (0.122)	0.301 (0.125)
cis	0.1	0.289 (0.104)	0.345 (0.108)	0.366 (0.1)	0.39 (0.098)	0.349 (0.109)	0.342 (0.113)
	0.15	0.272 (0.101)	0.329 (0.105)	0.36 (0.096)	0.396 (0.096)	0.373 (0.102)	0.361 (0.105)
	0.2	0.264 (0.098)	0.315 (0.103)	0.357 (0.094)	0.399 (0.095)	0.381 (0.099)	0.369 (0.099)
	0.25	0.263 (0.094)	0.305 (0.1)	0.359 (0.093)	0.402 (0.094)	0.384 (0.097)	0.374 (0.097)
Unweighted	0.05	-0.714 (0.065)	-0.65 (0.104)	0.03 (0.181)	-0.001 (0.017)	-0.105 (0.022)	-0.267 (0.082)
$trans$	0.1	-0.711 (0.036)	-0.729 (0.061)	-0.019 (0.17)	0.02 (0.015)	0.006 (0.016)	-0.433 (0.058)
	0.15	-0.67 (0.025)	-0.73 (0.039)	-0.073 (0.159)	0.031 (0.016)	0.025 (0.015)	-0.49 (0.053)
	0.2	-0.621 (0.019)	-0.703 (0.027)	-0.13 (0.148)	0.044 (0.018)	0.038 (0.017)	-0.501 (0.058)
	0.25	-0.57 (0.015)	-0.663 (0.02)	-0.187 (0.136)	0.062 (0.025)	0.053 (0.022)	-0.49 (0.069)
Weighted	0.05	-0.566 (0.077)	-0.429 (0.121)	0.32 (0.197)	-0.001 (0.017)	-0.104 (0.022)	-0.241 (0.083)
$trans$	0.1	-0.617 (0.045)	-0.573 (0.074)	0.287 (0.187)	0.02 (0.015)	0.006 (0.017)	-0.411 (0.058)
	0.15	-0.605 (0.031)	-0.612 (0.051)	0.244 (0.177)	0.03 (0.016)	0.025 (0.015)	-0.469 (0.052)
	0.2	-0.575 (0.024)	-0.613 (0.037)	0.191 (0.164)	0.042 (0.018)	0.036 (0.017)	-0.483 (0.057)
	0.25	-0.537 (0.019)	-0.595 (0.028)	0.135 (0.151)	0.059 (0.023)	0.051 (0.021)	-0.474 (0.067)

Table S 5. Correlation of gene-level degree with degree of PANDA and WGCNA networks by tissue. For each tissue, PANDA and WGCNA networks were construction and the gene-level degree was calculated. We then assessed the tissue-specific correlation of the PANDA and WGCNA gene-level degree with the proposed Benjamini-Hochberg (BH) degree, thresholded with $\tau = 0.05$ and weighted by the Z-statistic. Tissue-specific sample sizes (n) are provided. Related to STAR methods

Tissue	PANDA (n)	PANDA	WGCNA (n)	WGCNA
Adipose - subcutaneous	21078	0.075	21078	-0.004
Adipose - visceral	21068	0.049	21068	-0.018
Adrenal gland	19814	0.036	19814	-0.065
Artery - aorta	20386	0.087	20386	-0.045
Artery - coronary	19838	0.009	19838	-0.085
Artery - tibial	20141	0.135	20141	-0.014
Brain - cerebellum	20768	0.084	20768	-0.057
Brain - cortex	20647	0.020	20647	-0.012
Brain - nucleus accumbens basal ganglia	20234	-0.013	20234	-0.041
Cells - cultured fibroblasts	18957	0.121	18957	0.024
Colon - sigmoid	20709	0.031	20709	-0.070
Colon - transverse	21397	0.027	21397	-0.051
Esophagus - gastroesophageal junction	20453	0.024	20453	-0.031
Esophagus - mucosa	20643	0.082	20643	-0.036
Esophagus - muscularis	20551	0.049	20551	-0.020
Heart - atrial appendage	19894	0.036	19894	0.014
Heart - left ventricle	18554	0.031	18554	0.025
Liver	18147	-0.031	18147	-0.037
Lung	21948	0.039	21948	-0.026
Muscle Skeletal	18586	0.090	18586	0.015
Nerve - tibial	21626	0.109	21626	0.051
Pancreas	19421	0.004	19421	-0.068
Pituitary	21704	0.004	21704	-0.089
Skin - not sun exposed	21372	0.092	21372	-0.044
Skin - sun exposed	21475	0.097	21475	-0.034
Spleen	20901	0.026	20901	-0.065
Stomach	20618	0.002	20618	-0.054
Thyroid	21850	0.079	21850	0.0001
Whole blood	17731	0.059	17731	0.015

Table S 6. Meta-analysis correlation of gene-level degree with degree of PANDA or WGCNA network. For each of twenty-nine tissues, we estimated the correlation between the PANDA or WGCNA degree and the weighted BH degree across all threshold τ and then meta-analyzed. Related to STAR methods

	τ	Q-value	LFDR	BH
Unweighted PANDA	0.05	0.08 (0.07, 0.1)	0.07 (0.06, 0.08)	0.05 (0.04, 0.07)
	0.1	0.09 (0.08, 0.1)	0.08 (0.07, 0.09)	0.06 (0.04, 0.07)
	0.15	0.1 (0.09, 0.1)	0.08 (0.07, 0.09)	0.06 (0.05, 0.08)
	0.2	0.1 (0.09, 0.11)	0.09 (0.08, 0.1)	0.06 (0.05, 0.08)
	0.25	0.1 (0.09, 0.1)	0.09 (0.08, 0.1)	0.07 (0.05, 0.08)
Weighted PANDA	0.05	0.08 (0.07, 0.09)	0.06 (0.05, 0.07)	0.05 (0.04, 0.07)
	0.1	0.08 (0.07, 0.09)	0.07 (0.06, 0.08)	0.06 (0.04, 0.07)
	0.15	0.09 (0.08, 0.1)	0.07 (0.06, 0.08)	0.06 (0.04, 0.07)
	0.2	0.09 (0.08, 0.1)	0.08 (0.07, 0.09)	0.06 (0.05, 0.07)
	0.25	0.1 (0.09, 0.1)	0.08 (0.07, 0.09)	0.06 (0.05, 0.08)
Unweighted WGCNA	0.05	-0.02 (-0.03, -0.01)	-0.02 (-0.04, -0.01)	-0.02 (-0.04, -0.01)
	0.1	-0.02 (-0.03, -0.01)	-0.02 (-0.03, -0.01)	-0.02 (-0.04, -0.01)
	0.15	-0.02 (-0.03, -0.01)	-0.02 (-0.03, -0.01)	-0.02 (-0.03, -0.01)
	0.2	-0.01 (-0.02, 0)	-0.01 (-0.02, 0)	-0.02 (-0.03, -0.01)
	0.25	0 (-0.01, 0)	0 (-0.01, 0.01)	-0.02 (-0.03, -0.01)
Weighted WGCNA	0.05	-0.03 (-0.04, -0.01)	-0.03 (-0.04, -0.02)	-0.03 (-0.04, -0.02)
	0.1	-0.02 (-0.03, -0.01)	-0.02 (-0.03, -0.01)	-0.03 (-0.04, -0.01)
	0.15	-0.02 (-0.03, -0.01)	-0.02 (-0.03, -0.01)	-0.03 (-0.04, -0.01)
	0.2	-0.01 (-0.02, 0)	-0.01 (-0.02, 0)	-0.03 (-0.04, -0.01)
	0.25	0 (-0.01, 0.01)	0 (-0.01, 0.01)	-0.03 (-0.04, -0.01)

Table S 7. Correlation of gene-level degree with nucleotide diversity and Tajima's D by tissue. For each tissue, we assessed the tissue-specific correlation of gene-level nucleotide diveristy and Tajima's D statistic with the proposed Benjamini-Hochberg (BH) degree, thresholded with $\tau = 0.05$ and weighted by the Z-statistic. Tissue-specific sample sizes (n) are provided. Related to STAR methods

Tissue	Nucleotide diversity (n)	Nucleotide diversity	Tajima's D (n)	Tajima's D
Adipose - subcutaneous	19043	0.158	19043	0.157
Adipose - visceral	17561	0.136	17561	0.141
Adrenal gland	13373	0.106	13373	0.122
Artery - aorta	17159	0.160	17159	0.160
Artery - coronary	11762	0.083	11762	0.102
Artery - tibial	18258	0.179	18258	0.172
Brain - cerebellum	16368	0.132	16368	0.143
Brain - cortex	14277	0.100	14277	0.124
Brain - nucleus accumbens	12999	0.077	12999	0.111
Cells - cultured fibroblasts	17360	0.215	17360	0.195
Colon - sigmoid	15804	0.112	15804	0.126
Colon - transverse	16883	0.105	16883	0.124
Esophagus - gastroesophageal junction	15983	0.114	15983	0.124
Esophagus - mucosa	18310	0.179	18310	0.177
Esophagus - muscularis	18060	0.156	18060	0.160
Heart - atrial appendage	15928	0.141	15928	0.146
Heart - left ventricle	14330	0.134	14330	0.149
Liver	10403	0.088	10403	0.108
Lung	18963	0.143	18963	0.146
Muscle Skeletal	16532	0.160	16532	0.173
Nerve - tibial	20323	0.199	20323	0.188
Pancreas	14885	0.133	14885	0.143
Pituitary	15258	0.081	15258	0.098
Skin - not sun exposed	19077	0.157	19077	0.162
Skin - sun exposed	19755	0.162	19755	0.165
Spleen	15766	0.125	15766	0.139
Stomach	14539	0.084	14539	0.108
Thyroid	20519	0.175	20519	0.171
Whole blood	15743	0.169	15743	0.161

Table S 8. Meta-analysis correlation of gene-level degree with nucleotide diversity and Tajima's D. For each of twenty-nine tissues, we estimated the correlation between nucleotide diversity or Tajima's D and the weighted BH degree across all threshold τ and then meta-analyzed. Related to STAR methods

	τ	Q-value	LFDR	BH
Unweighted nucleotide diversity	0.05	0.19 (0.18, 0.2)	0.16 (0.15, 0.17)	0.14 (0.13, 0.16)
	0.1	0.19 (0.18, 0.2)	0.18 (0.17, 0.19)	0.16 (0.15, 0.18)
	0.15	0.19 (0.19, 0.2)	0.18 (0.17, 0.19)	0.17 (0.16, 0.19)
	0.2	0.2 (0.19, 0.2)	0.18 (0.18, 0.19)	0.18 (0.16, 0.19)
	0.25	0.19 (0.19, 0.2)	0.19 (0.18, 0.19)	0.18 (0.17, 0.2)
Weighted nucleotide diversity	0.05	0.18 (0.17, 0.19)	0.15 (0.14, 0.16)	0.14 (0.12, 0.15)
	0.1	0.18 (0.17, 0.19)	0.17 (0.16, 0.18)	0.16 (0.14, 0.17)
	0.15	0.18 (0.18, 0.19)	0.17 (0.16, 0.18)	0.17 (0.15, 0.18)
	0.2	0.19 (0.18, 0.2)	0.17 (0.16, 0.18)	0.17 (0.16, 0.19)
	0.25	0.19 (0.18, 0.2)	0.17 (0.17, 0.18)	0.18 (0.16, 0.19)
Unweighted Tajima's D	0.05	0.17 (0.16, 0.18)	0.15 (0.14, 0.16)	0.15 (0.14, 0.16)
	0.1	0.17 (0.16, 0.17)	0.16 (0.15, 0.17)	0.16 (0.15, 0.17)
	0.15	0.17 (0.16, 0.17)	0.16 (0.16, 0.17)	0.16 (0.15, 0.17)
	0.2	0.16 (0.16, 0.17)	0.16 (0.16, 0.17)	0.17 (0.16, 0.18)
	0.25	0.16 (0.16, 0.17)	0.16 (0.16, 0.17)	0.17 (0.16, 0.18)
Weighted Tajima's D	0.05	0.16 (0.16, 0.17)	0.15 (0.14, 0.16)	0.14 (0.13, 0.15)
	0.1	0.17 (0.16, 0.17)	0.16 (0.15, 0.17)	0.16 (0.15, 0.17)
	0.15	0.17 (0.16, 0.17)	0.16 (0.15, 0.17)	0.16 (0.15, 0.17)
	0.2	0.17 (0.16, 0.17)	0.16 (0.15, 0.17)	0.17 (0.16, 0.18)
	0.25	0.17 (0.16, 0.17)	0.16 (0.15, 0.17)	0.17 (0.16, 0.18)

Table S 9. Heritability analysis between SNP-level and gene-level degree and blood-related traits. We performed LD score regression to assess the enrichment of the SNP-level and gene-level Benjamini-Hochberg (BH) degree, thresholded with $\tau = 0.05$ and weighted by the Z-statistic, in blood-related traits and relevant tissues. Related to Figure 5

Tissue	Trait	SNP-level Enrichment (P)	SNP-level Coefficient (SE)	Gene-level Enrichment (P)	Gene-level Coefficient (SE)
Artery - aorta	Eosinophil	2.05 (5.92E-03)	7.64E-10 (1.64E-08)	1.32 (6.09E-03)	-6.35E-09 (5.63E-09)
Artery - aorta	Platelet	2.42 (6.62E-04)	3.40E-09 (2.89E-08)	1.56 (1.09E-06)	-5.77E-09 (9.24E-09)
Artery - aorta	RBC Width	3.29 (8.84E-06)	1.95E-08 (2.20E-08)	1.91 (1.55E-09)	4.97E-09 (7.16E-09)
Artery - aorta	Red Cell Count	2.47 (9.92E-06)	6.22E-09 (1.36E-08)	1.53 (2.95E-06)	-4.06E-10 (5.45E-09)
Artery - aorta	White Cell Count	2.02 (1.06E-04)	-2.18E-09 (1.11E-08)	1.34 (1.99E-05)	-5.68E-09 (3.71E-09)
Artery - aorta	HDL	2.09 (3.42E-03)	-2.08E-09 (8.85E-09)	1.26 (4.16E-02)	-7.04E-09 (3.90E-09)
Artery - aorta	LDL	2.09 (1.90E-02)	-9.84E-09 (9.64E-09)	1.72 (2.08E-04)	1.53E-09 (4.51E-09)
Artery - cor.	Eosinophil	1.53 (2.28E-01)	-1.53E-08 (1.88E-08)	1.37 (1.19E-02)	-4.54E-09 (6.60E-09)
Artery - cor.	Platelet	2.01 (8.59E-02)	-1.12E-08 (3.77E-08)	1.54 (1.75E-04)	-9.23E-09 (9.45E-09)
Artery - cor.	RBC Width	3.67 (4.25E-03)	3.79E-08 (3.69E-08)	2.25 (3.49E-08)	1.53E-08 (9.41E-09)
Artery - cor.	Red Cell Count	2.00 (7.65E-02)	-9.96E-09 (2.40E-08)	1.44 (1.27E-03)	-7.60E-09 (6.04E-09)
Artery - cor.	White Cell Count	1.73 (6.94E-02)	-6.78E-09 (1.65E-08)	1.32 (7.63E-04)	-6.65E-09 (4.09E-09)
Artery - cor.	HDL	1.57 (1.71E-01)	-1.26E-08 (9.90E-09)	1.27 (6.26E-02)	-6.83E-09 (3.90E-09)
Artery - cor.	LDL	1.47 (5.01E-01)	-1.84E-08 (1.33E-08)	1.84 (5.17E-03)	2.36E-09 (6.53E-09)
Artery - tibial	Eosinophil	2.21 (2.15E-04)	8.00E-09 (1.44E-08)	1.32 (2.21E-03)	-7.15E-09 (5.27E-09)
Artery - tibial	Platelet	2.67 (1.93E-06)	2.10E-08 (2.42E-08)	1.54 (9.91E-07)	-9.29E-09 (8.82E-09)
Artery - tibial	RBC Width	3.29 (1.11E-07)	2.12E-08 (1.87E-08)	1.88 (7.71E-08)	2.52E-09 (7.87E-09)
Artery - tibial	Red Cell Count	2.50 (4.55E-08)	8.53E-09 (1.14E-08)	1.31 (9.50E-04)	-1.57E-08 (4.50E-09)
Artery - tibial	White Cell Count	2.15 (1.77E-07)	3.74E-09 (9.58E-09)	1.31 (1.22E-05)	-7.75E-09 (3.59E-09)
Artery - tibial	HDL	2.03 (4.10E-04)	-4.14E-09 (7.30E-09)	1.31 (3.89E-03)	-4.94E-09 (3.23E-09)
Artery - tibial	LDL	2.17 (3.05E-03)	-9.20E-09 (9.06E-09)	1.57 (3.84E-03)	-2.44E-09 (5.06E-09)
Heart atr. app.	Eosinophil	2.19 (3.90E-03)	7.01E-09 (1.76E-08)	1.43 (3.37E-04)	-8.76E-10 (5.66E-09)
Heart atr. app.	Platelet	2.38 (2.18E-03)	-8.34E-11 (3.04E-08)	1.61 (6.74E-08)	-2.11E-09 (7.91E-09)
Heart atr. app.	RBC Width	3.73 (2.55E-06)	3.72E-08 (2.34E-08)	1.99 (7.72E-09)	6.58E-09 (7.55E-09)
Heart atr. app.	Red Cell Count	2.84 (7.35E-06)	2.55E-08 (1.74E-08)	1.43 (1.61E-04)	-7.08E-09 (5.71E-09)
Heart atr. app.	White Cell Count	2.21 (5.02E-04)	6.86E-09 (1.47E-08)	1.33 (3.67E-05)	-6.26E-09 (3.81E-09)
Heart atr. app.	HDL	2.26 (5.12E-03)	1.89E-09 (1.03E-08)	1.50 (4.61E-04)	9.00E-10 (4.08E-09)
Heart atr. app.	LDL	2.12 (2.56E-02)	-9.63E-09 (9.60E-09)	2.14 (5.74E-06)	1.16E-08 (5.17E-09)
Heart left vent.	Eosinophil	2.44 (2.33E-03)	1.74E-08 (2.02E-08)	1.49 (4.44E-05)	-1.97E-09 (5.50E-09)
Heart left vent.	Platelet	2.35 (3.03E-03)	-4.24E-09 (3.05E-08)	1.72 (5.26E-09)	-7.29E-10 (8.52E-09)
Heart left vent.	RBC Width	3.92 (1.25E-05)	4.25E-08 (2.73E-08)	2.12 (1.18E-08)	7.69E-09 (8.50E-09)
Heart left vent.	Red Cell Count	2.84 (2.51E-05)	2.26E-08 (1.88E-08)	1.48 (8.58E-05)	-1.01E-08 (6.04E-09)
Heart left vent.	White Cell Count	2.26 (7.55E-04)	7.91E-09 (1.61E-08)	1.51 (6.92E-08)	-5.96E-10 (4.34E-09)
Heart left vent.	HDL	2.14 (7.31E-03)	-2.54E-09 (1.02E-08)	1.48 (2.67E-03)	-2.61E-09 (4.42E-09)
Heart left vent.	LDL	2.34 (1.92E-02)	-5.86E-09 (1.10E-08)	2.23 (2.06E-06)	1.11E-08 (5.21E-09)
Whole blood	Eosinophil	3.04 (1.50E-05)	2.62E-08 (1.98E-08)	1.86 (3.75E-08)	1.70E-09 (7.36E-09)
Whole blood	Platelet	3.19 (1.84E-07)	4.28E-08 (2.74E-08)	1.99 (2.33E-10)	6.89E-09 (1.16E-08)
Whole blood	RBC Width	4.25 (2.06E-09)	5.20E-08 (2.13E-08)	2.45 (2.25E-11)	1.58E-08 (9.03E-09)
Whole blood	Red Cell Count	3.03 (1.01E-06)	2.11E-08 (1.93E-08)	1.71 (3.87E-08)	-5.90E-09 (6.80E-09)
Whole blood	White Cell Count	2.83 (1.36E-08)	2.07E-08 (1.35E-08)	1.74 (2.41E-11)	5.22E-10 (5.15E-09)
Whole blood	HDL	2.58 (1.49E-05)	3.20E-09 (8.51E-09)	1.65 (3.06E-03)	-2.52E-09 (7.15E-09)
Whole blood	LDL	2.38 (4.34E-03)	-9.41E-09 (1.05E-08)	1.81 (6.48E-04)	-3.97E-09 (5.36E-09)

Table S 10. Heritability analysis between SNP-level and gene-level degree and blood-related traits. We performed LD score regression to assess the enrichment of the SNP-level and gene-level Q-value (QV) degree, thresholded with $\tau = 0.05$ and weighted by the Z-statistic, in blood-related traits and relevant tissues. Related to Figure 5

Tissue	Trait	SNP-level Enrichment (P)	SNP-level Coefficient (SE)	Gene-level Enrichment (P)	Gene-level Coefficient (SE)
Artery - aorta	Eosinophil	2.05 (1.59E-03)	3.31E-09 (1.46E-08)	1.30 (3.05E-03)	-6.67E-09 (5.22E-09)
Artery - aorta	Platelet	2.35 (3.49E-05)	3.61E-09 (2.35E-08)	1.57 (1.93E-09)	-1.20E-09 (8.12E-09)
Artery - aorta	RBC Width	3.04 (8.32E-07)	1.55E-08 (1.81E-08)	1.79 (1.84E-11)	2.61E-09 5.40E-09)
Artery - aorta	Red Cell Count	2.43 (6.79E-08)	9.59E-09 (1.11E-08)	1.45 (4.77E-07)	-3.86E-09 (4.51E-09)
Artery - aorta	White Cell Count	2.01 (3.34E-06)	1.32E-10 (9.42E-09)	1.33 (1.31E-06)	-5.29E-09 (3.35E-09)
Artery - aorta	HDL	1.96 (1.87E-03)	-3.70E-09 (7.86E-09)	1.24 (2.45E-02)	-6.56E-09 (3.61E-09)
Artery - aorta	LDL	2.26 (5.99E-03)	-4.00E-09 (1.00E-08)	1.60 (2.28E-04)	-2.22E-11 (3.90E-09)
Artery - cor.	Eosinophil	1.34 (3.40E-01)	-1.99E-08 (1.60E-08)	1.32 (3.01E-04)	-3.63E-09 (4.32E-09)
Artery - cor.	Platelet	1.84 (3.89E-02)	-1.25E-08 (2.74E-08)	1.43 (1.39E-07)	-1.08E-08 (6.45E-09)
Artery - cor.	RBC Width	2.77 (1.82E-03)	1.51E-08 (2.36E-08)	1.86 (1.50E-10)	7.80E-09 (5.63E-09)
Artery - cor.	Red Cell Count	1.87 (1.09E-02)	-7.34E-09 (1.49E-08)	1.37 (5.34E-05)	-8.36E-09 (4.48E-09)
Artery - cor.	White Cell Count	1.63 (3.00E-02)	-7.07E-09 (1.24E-08)	1.36 (1.45E-08)	-2.39E-09 (3.16E-09)
Artery - cor.	HDL	1.46 (1.43E-01)	-1.03E-08 (8.37E-09)	1.38 (4.72E-03)	-1.48E-09 (4.12E-09)
Artery - cor.	LDL	1.52 (3.13E-01)	-1.33E-08 (1.04E-08)	1.62 (5.33E-04)	9.74E-10 (4.42E-09)
Artery - tibial	Eosinophil	2.08 (2.78E-04)	3.87E-09 (1.35E-08)	1.26 (5.54E-03)	-9.86E-09 (4.82E-09)
Artery - tibial	Platelet	2.56 (6.00E-08)	1.86E-08 (2.05E-08)	1.52 (4.01E-07)	-7.87E-09 (8.79E-09)
Artery - tibial	RBC Width	3.08 (3.81E-08)	1.74E-08 (1.71E-08)	1.78 (2.87E-08)	5.67E-10 (6.95E-09)
Artery - tibial	Red Cell Count	2.36 (8.69E-09)	5.52E-09 (1.02E-08)	1.30 (4.07E-04)	-1.40E-08 (4.45E-09)
Artery - tibial	White Cell Count	2.12 (1.03E-08)	4.38E-09 (8.80E-09)	1.32 (4.47E-06)	-6.25E-09 (3.59E-09)
Artery - tibial	HDL	1.95 (1.87E-04)	-4.90E-09 (6.52E-09)	1.27 (7.43E-03)	-5.26E-09 (3.23E-09)
Artery - tibial	LDL	2.55 (3.81E-04)	2.36E-09 (9.46E-09)	1.51 (2.14E-03)	-2.69E-09 (4.29E-09)
Heart atr. app.	Eosinophil	2.12 (1.09E-03)	6.83E-09 (1.49E-08)	1.41 (1.02E-04)	2.97E-10 (5.00E-09)
Heart atr. app.	Platelet	2.42 (3.98E-05)	9.41E-09 (2.42E-08)	1.58 (5.39E-12)	1.09E-09 (5.70E-09)
Heart atr. app.	RBC Width	3.36 (1.66E-07)	2.99E-08 (1.88E-08)	1.82 (1.58E-09)	4.50E-09 (6.40E-09)
Heart atr. app.	Red Cell Count	2.57 (3.38E-07)	1.76E-08 (1.37E-08)	1.38 (2.69E-05)	-8.06E-09 (4.73E-09)
Heart atr. app.	White Cell Count	2.04 (8.84E-05)	1.76E-09 (1.16E-08)	1.34 (7.42E-07)	-4.30E-09 (3.29E-09)
Heart atr. app.	HDL	2.13 (7.74E-04)	1.33E-09 (7.87E-09)	1.37 (1.72E-03)	-1.37E-09 (3.51E-09)
Heart atr. app.	LDL	1.92 (2.22E-02)	-1.23E-08 (8.29E-09)	1.76 (1.76E-05)	4.84E-09 (3.87E-09)
Heart left vent.	Eosinophil	2.24 (8.04E-04)	1.12E-08 (1.64E-08)	1.43 (1.61E-05)	-3.07E-09 (4.72E-09)
Heart left vent.	Platelet	2.42 (3.01E-05)	6.28E-09 (2.38E-08)	1.70 (3.60E-10)	4.12E-09 (8.27E-09)
Heart left vent.	RBC Width	3.40 (8.94E-07)	2.91E-08 (2.09E-08)	1.96 (1.85E-11)	6.31E-09 (6.37E-09)
Heart left vent.	Red Cell Count	2.69 (1.93E-07)	2.10E-08 (1.43E-08)	1.53 (1.68E-07)	-3.40E-09 (5.07E-09)
Heart left vent.	White Cell Count	2.30 (1.43E-05)	1.24E-08 (1.34E-08)	1.44 (6.98E-09)	-2.51E-09 (3.65E-09)
Heart left vent.	HDL	2.14 (5.18E-04)	1.77E-10 (7.73E-09)	1.57 (2.95E-04)	2.11E-09 (5.28E-09)
Heart left vent.	LDL	2.21 (5.74E-03)	-6.69E-09 (8.59E-09)	1.98 (6.61E-07)	7.80E-09 (3.95E-09)
Whole blood	Eosinophil	2.80 (1.18E-05)	1.96E-08 (1.78E-08)	1.85 (1.39E-09)	2.96E-09 (6.52E-09)
Whole blood	Platelet	2.90 (1.03E-08)	2.98E-08 (2.24E-08)	1.93 (3.04E-12)	5.16E-09 (9.83E-09)
Whole blood	RBC Width	3.92 (1.58E-10)	4.63E-08 (1.88E-08)	2.26 (2.66E-12)	9.29E-09 (7.95E-09)
Whole blood	Red Cell Count	2.94 (9.40E-09)	2.49E-08 (1.62E-08)	1.63 (6.87E-09)	-9.90E-09 (5.86E-09)
Whole blood	White Cell Count	2.70 (1.22E-10)	1.95E-08 (1.12E-08)	1.70 (6.58E-13)	-8.12E-10 (4.66E-09)
Whole blood	HDL	2.55 (1.90E-06)	6.10E-09 (8.25E-09)	1.59 (1.84E-03)	-3.89E-09 (6.58E-09)
Whole blood	LDL	2.70 (3.31E-04)	1.38E-09 (1.01E-08)	1.86 (6.41E-05)	-1.20E-09 (5.12E-09)

Table S 11. Heritability analysis between SNP-level and gene-level degree and blood-related traits. We performed LD score regression to assess the enrichment of the SNP-level and gene-level LFDR degree, thresholded with $\tau = 0.05$ and weighted by the Z-statistic, in blood-related traits and relevant tissues. Related to Figure 5

Tissue	Trait	SNP-level Enrichment (P)	SNP-level Coefficient (SE)	Gene-level Enrichment (P)	Gene-level Coefficient (SE)
Artery - aorta	Eosinophil	2.12 (2.76E-03)	5.88E-09 (1.59E-08)	1.34 (1.07E-03)	-6.63E-09 (5.28E-09)
Artery - aorta	Platelet	2.40 (5.96E-04)	4.81E-09 (2.83E-08)	1.65 (3.66E-10)	2.36E-09 (8.50E-09)
Artery - aorta	RBC Width	3.20 (1.24E-05)	1.78E-08 (2.15E-08)	1.84 (4.30E-11)	2.26E-09 (5.92E-09)
Artery - aorta	Red Cell Count	2.38 (1.74E-05)	3.88E-09 (1.33E-08)	1.47 (6.68E-07)	-4.99E-09 (4.66E-09)
Artery - aorta	White Cell Count	2.04 (4.03E-05)	3.89E-10 (1.07E-08)	1.37 (1.61E-07)	-4.82E-09 (3.48E-09)
Artery - aorta	HDL	2.01 (3.35E-03)	-3.53E-09 (8.26E-09)	1.24 (3.73E-02)	-7.65E-09 (3.90E-09)
Artery - aorta	LDL	2.06 (1.81E-02)	-9.75E-09 (9.38E-09)	1.62 (5.24E-04)	-7.66E-10 (4.10E-09)
Artery - cor.	Eosinophil	1.43 (3.14E-01)	-1.72E-08 (1.85E-08)	1.33 (1.45E-03)	-5.54E-09 (5.05E-09)
Artery - cor.	Platelet	1.86 (8.26E-02)	-1.53E-08 (3.27E-08)	1.52 (2.73E-08)	-6.83E-09 (6.94E-09)
Artery - cor.	RBC Width	3.06 (5.35E-03)	2.00E-08 (2.96E-08)	1.93 (2.39E-12)	6.88E-09 (5.39E-09)
Artery - cor.	Red Cell Count	1.90 (5.04E-02)	-1.04E-08 (1.96E-08)	1.43 (6.95E-06)	-7.14E-09 (4.67E-09)
Artery - cor.	White Cell Count	1.69 (5.66E-02)	-6.01E-09 (1.50E-08)	1.38 (1.38E-08)	-3.27E-09 (3.44E-09)
Artery - cor.	HDL	1.36 (3.29E-01)	-1.52E-08 (9.16E-09)	1.35 (2.42E-02)	-3.74E-09 (4.84E-09)
Artery - cor.	LDL	1.23 (7.00E-01)	-2.13E-08 (1.19E-08)	1.61 (1.83E-03)	-1.27E-09 (4.71E-09)
Artery - tibial	Eosinophil	2.22 (2.44E-04)	9.97E-09 (1.44E-08)	1.29 (2.28E-03)	-9.72E-09 (4.91E-09)
Artery - tibial	Platelet	2.59 (6.73E-06)	1.78E-08 (2.45E-08)	1.59 (1.17E-08)	-4.73E-09 (8.27E-09)
Artery - tibial	RBC Width	3.28 (1.92E-07)	2.25E-08 (1.88E-08)	1.85 (7.16E-09)	1.21E-09 (7.27E-09)
Artery - tibial	Red Cell Count	2.46 (1.31E-07)	8.35E-09 (1.14E-08)	1.31 (2.52E-04)	-1.60E-08 (4.22E-09)
Artery - tibial	White Cell Count	2.11 (7.01E-07)	2.75E-09 (9.62E-09)	1.32 (2.74E-06)	-7.67E-09 (3.63E-09)
Artery - tibial	HDL	1.97 (7.74E-04)	-4.89E-09 (7.35E-09)	1.31 (1.56E-03)	-5.04E-09 (3.15E-09)
Artery - tibial	LDL	2.10 (5.09E-03)	-9.77E-09 (9.07E-09)	1.54 (3.30E-03)	-3.26E-09 (4.88E-09)
Heart atr. app.	Eosinophil	2.20 (3.75E-03)	9.31E-09 (1.78E-08)	1.37 (3.03E-04)	-5.01E-09 (4.86E-09)
Heart atr. app.	Platelet	2.29 (2.26E-03)	-3.10E-09 (2.90E-08)	1.59 (1.73E-11)	-3.03E-09 (5.86E-09)
Heart atr. app.	RBC Width	3.60 (4.27E-06)	3.50E-08 (2.29E-08)	1.92 (7.51E-10)	5.03E-09 (6.78E-09)
Heart atr. app.	Red Cell Count	2.74 (7.15E-06)	2.29E-08 (1.65E-08)	1.40 (2.64E-05)	-9.96E-09 (4.94E-09)
Heart atr. app.	White Cell Count	2.17 (3.94E-04)	6.90E-09 (1.41E-08)	1.37 (8.75E-08)	-5.40E-09 (3.35E-09)
Heart atr. app.	HDL	2.22 (3.64E-03)	2.27E-09 (9.62E-09)	1.45 (5.64E-04)	-7.04E-10 (3.74E-09)
Heart atr. app.	LDL	1.98 (3.63E-02)	-1.16E-08 (9.13E-09)	1.93 (5.65E-05)	7.07E-09 (5.01E-09)
Heart left vent.	Eosinophil	2.45 (1.35E-03)	1.94E-08 (1.93E-08)	1.45 (1.17E-05)	-3.97E-09 (4.75E-09)
Heart left vent.	Platelet	2.45 (7.26E-04)	5.14E-09 (2.89E-08)	1.67 (3.87E-11)	-2.51E-09 (7.29E-09)
Heart left vent.	RBC Width	3.73 (1.18E-05)	3.75E-08 (2.57E-08)	2.07 (1.15E-10)	8.81E-09 (7.55E-09)
Heart left vent.	Red Cell Count	2.79 (1.44E-05)	2.20E-08 (1.78E-08)	1.47 (1.02E-06)	-9.67E-09 (4.95E-09)
Heart left vent.	White Cell Count	2.31 (2.45E-04)	1.14E-08 (1.55E-08)	1.48 (2.67E-09)	-2.36E-09 (3.87E-09)
Heart left vent.	HDL	2.14 (4.00E-03)	-1.32E-09 (9.31E-09)	1.62 (1.87E-04)	2.15E-09 (5.34E-09)
Heart left vent.	LDL	2.28 (1.91E-02)	-6.32E-09 (1.05E-08)	2.06 (4.56E-07)	8.17E-09 (4.05E-09)
Whole blood	Eosinophil	3.02 (1.62E-05)	2.71E-08 (1.97E-08)	1.85 (1.42E-09)	2.22E-09 (6.70E-09)
Whole blood	Platelet	3.13 (4.14E-07)	4.15E-08 (2.75E-08)	2.01 (4.76E-12)	1.10E-08 (1.09E-08)
Whole blood	RBC Width	4.24 (2.21E-09)	5.39E-08 (2.12E-08)	2.40 (1.49E-13)	1.53E-08 (8.04E-09)
Whole blood	Red Cell Count	2.99 (1.28E-06)	2.13E-08 (1.92E-08)	1.69 (2.81E-09)	-6.81E-09 (6.22E-09)
Whole blood	White Cell Count	2.85 (1.28E-08)	2.29E-08 (1.35E-08)	1.73 (1.04E-13)	2.62E-10 (4.74E-09)
Whole blood	HDL	2.60 (1.61E-05)	4.89E-09 (8.68E-09)	1.66 (8.93E-04)	-2.23E-09 (6.63E-09)
Whole blood	LDL	2.37 (3.78E-03)	-8.55E-09 (1.03E-08)	1.92 (1.72E-05)	-6.61E-10 (4.86E-09)

Table S 12. Computational cost across network construction and degree calculation approaches given for SNPs across all thresholds τ. Related to STAR methods

Step	Approach	Average time (sd)	Output size (.Rds)
1	Calculate edges for thresholded, location-specific methods.	154.9 (12.8) s	482 KB
	Calculate edges for exhaustive, genome-wide methods.	1104.4 (80.9) s	2093 KB
2	Calculate BH-based SNP degrees.	0.2 (0.03) s	347 KB
	Calculate QV-based SNP degrees.	0.4 (0.02) s	537 KB
	Calculate LFDR-based SNP degrees.	0.3 (0.02) s	401 KB
	Calculate NP-based SNP degrees.	0.1 (0.01) s	161 KB
