# Supplementary material for: Connectivity in eQTL networks dictates reproducibility and genomic properties
Source: Cell Rep Methods. 2022 May 23;2(5):100218. doi: 10.1016/j.crmeth.2022.100218 (PMC9142682; doi:10.1016/j.crmeth.2022.100218)
Supplement: Document S2. Article plus supplemental information [file mmc2.pdf]

Cell Reports Methods

Resource

Connectivity in eQTL networks dictates reproducibility and genomic properties

Graphical abstract

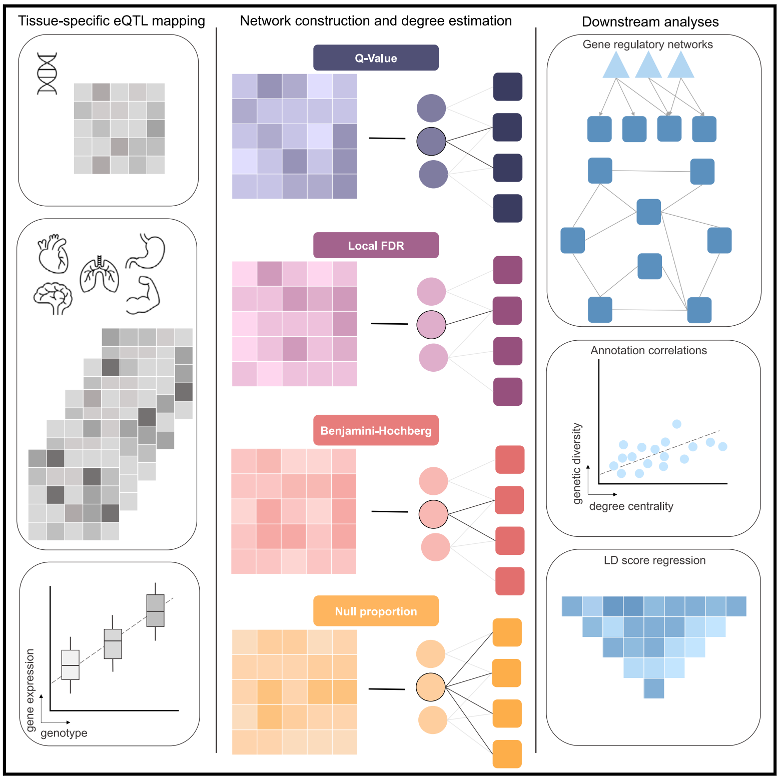

Authors

Sheila M. Gaynor, Maud Fagny, Xihong Lin, John Platig, John Quackenbush

Correspondence

johnq@hsph.harvard.edu

In brief

Networks provide a way to integratively characterize large collections of genomic associations. To assess the robustness of eQTL networks, Gaynor et al. present an extensive evaluation of network and metric specifications. They present a robust method for network edge definition and show its value in downstream gene- and trait-based analyses.

Highlights

- Bipartite networks can informatively represent sets of eQTL associations
- Weighted network definitions balance computational efficiency and stability
- Well-defined eQTL networks correlate with gene networks and genetic diversity
- Tissue-specific network metrics like degree are enriched for tissue-relevant traits

Gaynor et al., 2022, Cell Reports Methods 2, 100218
May 23, 2022 © 2022 The Author(s).
https://doi.org/10.1016/j.crmeth.2022.100218

CellPress

Cell Reports Methods

CellPress
OPEN ACCESS

Resource

Connectivity in eQTL networks dictates reproducibility and genomic properties

Sheila M. Gaynor,[1,2] Maud Fagny,[2,3] Xihong Lin,[1,4] John Platig,[2,5,6] and John Quackenbush[1,2,5,7,*]
[1]Department of Biostatistics, Harvard T.H. Chan School of Public Health, Boston, MA 02115, USA
[2]Department of Biostatistics and Computational Biology and Center for Cancer Computational Biology, Dana-Farber Cancer Institute, Boston, MA 02115, USA
[3]Université Paris-Saclay, INRAE, CNRS, AgroParisTech, GQE - Le Moulon, 91190 Gif-sur-Yvette, France
[4]Department of Statistics, Harvard University, Cambridge, MA 02138, USA
[5]Channing Division of Network Medicine, Brigham and Women's Hospital, Boston, MA 02115, USA
[6]Department of Medicine, Harvard Medical School, Boston, MA 02115, USA
[7]Lead contact
*Correspondence: johnq@hsph.harvard.edu
https://doi.org/10.1016/j.crmeth.2022.100218

MOTIVATION Expression quantitative trait locus (eQTL) analysis has been transformed by the introduction of network representations of eQTL SNP-gene associations. However, these have relied on using sometimes arbitrary thresholds to turn functions of these associations such as false discovery rates into binary presence/absence representations. We sought to identify optimal methods for defining eQTL networks and metrics that balance statistical robustness, computational complexity, and biological discovery.

SUMMARY

Expression quantitative trait locus (eQTL) analysis associates SNPs with gene expression; these relationships can be represented as a bipartite network with association strength as "edge weights" between SNPs and genes. However, most eQTL networks use binary edge weights based on thresholded FDR estimates: definitions that influence reproducibility and downstream analyses. We constructed twenty-nine tissue-specific eQTL networks using GTEx data and evaluated a comprehensive set of network specifications based on false discovery rates, test statistics, and p values, focusing on the degree centrality—a metric of an SNP or gene node's potential network influence. We found a thresholded Benjamini-Hochberg q value weighted by the Z-statistic balances metric reproducibility and computational efficiency. Our estimated gene degrees positively correlate with gene degrees in gene regulatory networks, demonstrating that these networks are complementary in understanding regulation. Gene degrees also correlate with genetic diversity, and heritability analyses show that highly connected nodes are enriched for tissue-relevant traits.

INTRODUCTION

Most human traits and diseases are influenced by many genetic variants that act in concert to alter cellular function (Hawkins et al., 2010). Experimental evidence has demonstrated that the overwhelming majority of trait-associated variants are enriched within regulatory elements (Albert and Kruglyak, 2015; Ward and Kellis, 2012; GTEx Consortium, 2015). Expression quantitative trait loci (eQTL), which associate genetic variants with gene expression, yield a substantial over-representation of genome-wide association study (GWAS) variants as eQTLs relative to expectation (Morley et al., 2004; Cheung et al., 2005; Schadt et al., 2005; Nicolae et al., 2010). This suggests that eQTLs play an important role in the causal pathway between genetic variants and phenotype, and it is further evidenced that tissue-specific disease-linked eQTLs are enriched in relevant tissues (Dermitzakis, 2008; Fagny et al., 2017). Common approaches to genetic and genomic analyses such as eQTL mapping consider only pairwise associations, failing to elucidate the molecular mechanisms by which multiple genetic variants relate to expression across genes (Ward and Kellis, 2012; Korte and Farlow, 2013). Integrative analyses of the complex relationships between genetic and genomic features that are reproducible and accurately represent biological relationships are thus of increasingly significant importance.

Network analyses provide an integrative approach to characterize complex genomic associations (Barabási et al., 2011). We can identify genetic variants and genes that collectively influence cellular processes to drive phenotypes using networks (Platig et al., 2016; Fagny et al., 2017). Bipartite networks naturally

This is an open access article under the CC BY license (http://creativecommons.org/licenses/by/4.0/).

represent eQTL associations, where the edges between SNPs and gene expression indicate the eQTL association (Asratian et al., 1998; Platig et al., 2016; Fagny et al., 2017; Barber, 2007). Well-defined features of a network can elucidate genetic regulation and inform function. The degree is a measure of network centrality that is associated with how essential a node is to function. For example, nodes that are more densely connected can represent natural divisions of functional relatedness. This representation has been shown to identify biological effects in chronic obstructive pulmonary disease (COPD) (Glass et al., 2014), where GWAS-identified SNPs were most central among groups of functionally related features (Platig et al., 2016; Fagny et al., 2017; Cho et al., 2014; Nicolae et al., 2010).

Many network approaches have been introduced to model genetic and genomic data. Such approaches often use methods like correlation and association-based measures to define networks; other approaches, including Bayesian network analysis, infer directed acyclic graphs to model causal effects from observational data (Zhu et al., 2004, 2012; Yazdani et al., 2016; Sedgewick et al., 2019; Badsha and Fu, 2019). Some approaches permit the use of many data types (such as multiomics data), incorporate prior evidence in modeling, and may allow missing data (Howey et al., 2021). Network methodologies vary in the assumptions made, often using multivariate distributional assumptions in order to obtain conditional dependencies between nodes. These assumptions may be violated in various settings, such as genetics settings with pleiotropy (Howey et al., 2020), and complex models often cannot operate on summary statistics. A straightforward approach is to define sets of associations directly as networks and permit them to represent a large graph from which one can perform secondary analyses, such as community detection, while maintaining the complexity of the associations (Platig et al., 2016).

Existing eQTL network analyses in particular have constructed networks using thresholded estimates from the eQTL analysis regressing gene expression on genotype (Albert and Kruglyak, 2015). This approach desirably imposes a small computational burden, as the network is limited to the sparse set of edges meeting a threshold but requires an informed threshold and selection of association measure, often selected in a semiquantitative manner. Reducing eQTL associations to such indicators to build a network may be detrimental by discarding potentially valuable data, detracting from potential reproducibility, and ultimately limiting the ability to perform informative downstream analyses. Methods that are more robust than dichotimization or based on rigorously defined measures or thresholds may overcome these limitations (Zhu et al., 2004, 2012; Yazdani et al., 2016; Sedgewick et al., 2019; Badsha and Fu, 2019). However, approaches that include fully weighted networks or that cannot operate on summary statistics have greater computational burden, given the need to retain and operate on output from millions of regression models, and do not necessarily ensure improved biological insight. It is thus critical to comprehensively evaluate potential network specifications in order to fully characterize eQTL network degrees, indicate robustness of network-based findings, and provide further biological insight.

In this article, we consider a comprehensive set of network representations of the SNP-gene association specifically toward estimating degree, a measure of how central a node is within the network. In an analysis of twenty-nine tissues from the Genotype-Tissue Expression (GTEx, v.8 release) project, we construct eQTL networks and estimate degree metrics for nodes. We consider definitions that vary with respect to estimation method, model output retained, threshold in dichotomized settings, and inclusion of weights. We evaluate the network reproducibility by considering consistency of degree in split-sample tissue-specific networks and across tissues for tissue-specific networks. Given our network characterization, which balances stability of SNP and gene degree with computation efficiency, we investigate the relationship of gene degree in the eQTL network to two other gene network types, gene regulatory networks and gene co-expression networks. We then consider the biological informativeness of the tissue-specific eQTL networks by evaluating the relationship of gene degree to genetic diversity measures and heritability enrichment of blood traits. Our results demonstrate that the topology of well-defined eQTL networks relates genomic features to genetic diversity and trait heritability.

RESULTS

eQTL networks are dependent on edge and degree definition

We mapped eQTLs from the genotype and RNA sequencing (RNA-seq) data for twenty-nine tissues (sample size n = 202–706) from the GTEx v.8 dataset (https://gtexportal.org/home/datasets). After data processing primarily to impute variants and normalize expression data, we retained 5,339,781 SNPs for all observations and 24,138 genes, on average, across tissues. We performed exhaustive eQTL mapping, adjusting for sex, genotyping platform and protocol, PEER factors, and the first five principal components by tissue (STAR Methods; Table S1); *cis*-eQTL mapping was performed for variants within 1 Mb of a gene's annotated transcriptional start site. We identified 806,182 (liver) to 3,930,834 (thyroid) *cis*-eQTLs and 18,037 (liver) and 110,952 (thyroid) *trans*-eQTLs at a false discovery rate (FDR) threshold of 0.05. We compared our *cis*-eQTL associations with those reported by the GTEx Consortium; on average, 79% (SD = 2%) of our *cis*-eQTLs were also in the GTEx results. These differences may be attributable to the genotype preparation and analysis methods.

We constructed eQTL networks from these results based on edge definitions varying in sparsity, estimation method, and weighting (Figure 1). The "sparse" representation includes edges where associations met a measure of significance below a threshold, τ, where τ was set equal to 0.05, 0.1, 0.15, and 0.2. These measures included the q value (QV) as defined by Storey et al. (Storey, 2002; Storey et al., 2003; Storey and Tibshirani, 2003), the local FDR (LFDR), and an adaptation of the Benjamini-Hochberg (BH) procedure for calculating the QV (see STAR Methods). These methods differ in their computational efficiency and probabilistic interpretation. We extended these unweighted sparse networks to be weighted by the eQTL Z-statistic to provide a measure of the strength of association. We also considered a so-called "denser" network representation that includes edges defined by the p value for all tested associations. p values permit natural definitions of network

CellPress
OPEN ACCESS

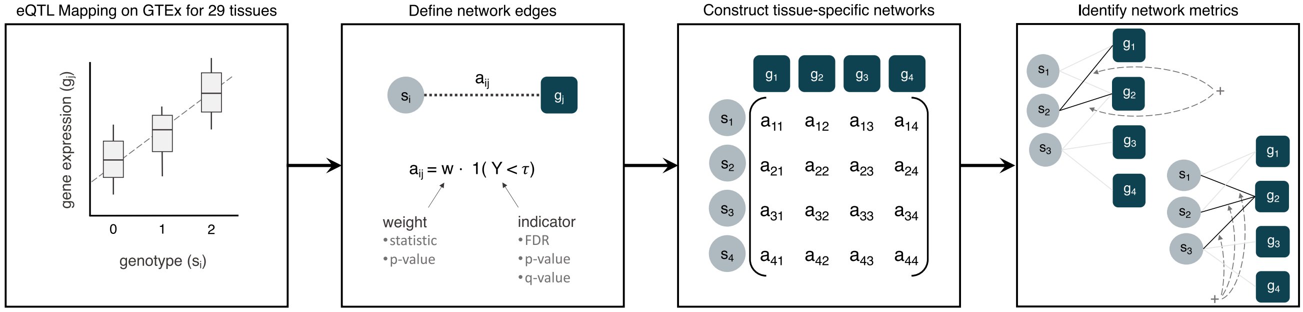

Figure 1. eQTL network workflow

eQTLs are mapped from genetic and gene expression data, and a function of their associations is used to construct an adjacency matrix with elements a_{ij}, from which network metrics such as degree can be calculated (for example, by edge summation) and used to infer scientific conclusions.

metrics. Networks were constructed for SNP-gene pairings on both a genome-wide and location-specific scale; in the genome-wide setting, edges are defined without regard to location, whereas the location-specific setting distinguished between *cis* and *trans* effects. Given the variety of methods for defining eQTL weights, we calculated degree metrics using definitions appropriate for the choice of edge weight. In the sparse settings, the degree was defined as the summation of all edges connected to a node, as is standard; for the denser representation, the degree was defined as the proportion of non-null proportion (NP) edges connected to a node. We calculated the degree metrics for the location-specific networks. In the sparse setting, we calculated the degree metrics for networks of all SNP-gene pairings from both the genome-wide and location-specific networks; the dense representation only permitted a genome-wide degree when considering all SNP-gene pairings.

Most association tests did not result in network SNP-gene edges (Figure 2), as expected given the relatively small number of eQTLs across the genome. The number of edges in the sparse networks generally decreased with decreasing sample size, as expected given corresponding decreasing power. The BH-based network consistently had fewer edges, followed by the LFDR-based network. The sparse networks had more non-zero edges when relaxing the threshold τ, as evidenced by the lighter shades within stacked bars in Figure 2. This is to be expected because it permits more "suggestive" associations as edges beyond those meeting more rigorous criteria. The NP-defined network includes edges for all associations by definition.

The distributions of the SNP degree across tissues where τ = 0.05 and edges are weighted by the magnitude of the eQTL Z-statistic are given in Figure 2. The distributions are right skewed, indicating that most SNPs have few connections and few SNPs are associated with many genes or with high effect size; this trend is observed for all τ as well as for gene degree. The unweighted degree estimates are less right skewed given that the strength of association is not incorporated to further inform the integer-valued degree. The unweighted-degree estimates are highly correlated with the weighed degree (STAR Methods; Table S2). The NP method yields probabilistic degrees for all nodes, most of which were estimated to be near or equal to zero. Consistent with previous findings that *cis*-eQTLs are more commonly identified than *trans*-eQTLs due to multiple testing challenges, the majority of SNP-gene edges are *cis*-eQTLs. The *cis* component constitutes 100% of the degree magnitude for most nodes, as variants with regulatory functions tend to act locally (within the megabase window used to define *cis* associations).

Degree definition determines stability

Within-tissue reproducibility

The stability of eQTL networks and their metrics is dependent on network definition and sufficient sample size. The reproducibility of degree estimates in independent and reduced samples were evaluated by computing the SNP and gene degree metrics for random sample splits and assessing the concordance between the split-sample degree estimates via Spearman correlation. This was repeated and averaged across five subsamples for the entire network (accounting for location in the sparse networks) and was restricted to *cis* and *trans* associations to account for variability (Figure 3; Table S3).

Of the sparse networks, the BH-based network had the highest average SNP degree correlations between sample splits for the tissues (τ = 0.05; unweighted median: 0.19, interquartile range [IQR]: 0.22; weighted median: 0.44, IQR: 0.19). The LFDR- (τ = 0.05; unweighted median: -0.02, IQR: 0.33; weighted median: 0.14, IQR: 0.36) and QV-based networks (τ = 0.05; unweighted median: -0.10, IQR: 0.34; weighted median: −0.003, IQR: 0.36) had lower average sample-split correlations for SNP degree. Each of the thresholded degree definitions demonstrated higher correlations for the weighted measures, which allows for greater granularity in the degree distribution. The average degree correlation between the splits decreases with increasing threshold τ for most definitions. A relaxed threshold may introduce more edges than can be stably estimated due to larger errors. The NP-based degree in the network of all eQTLs had averaged sample-split correlations with median -0.34 (IQR: 0.004) for SNP degree. This more dense network representation was not positively correlated across sample splits in any tissue. This is likely attributable to the instability of estimating null proportions among a large set of primarily null tests (Storey, 2002; Storey et al., 2003; Storey and Tibshirani, 2003) and a general lack of spread in the degree. The results for gene degree were notably more consistent across methods, which may be

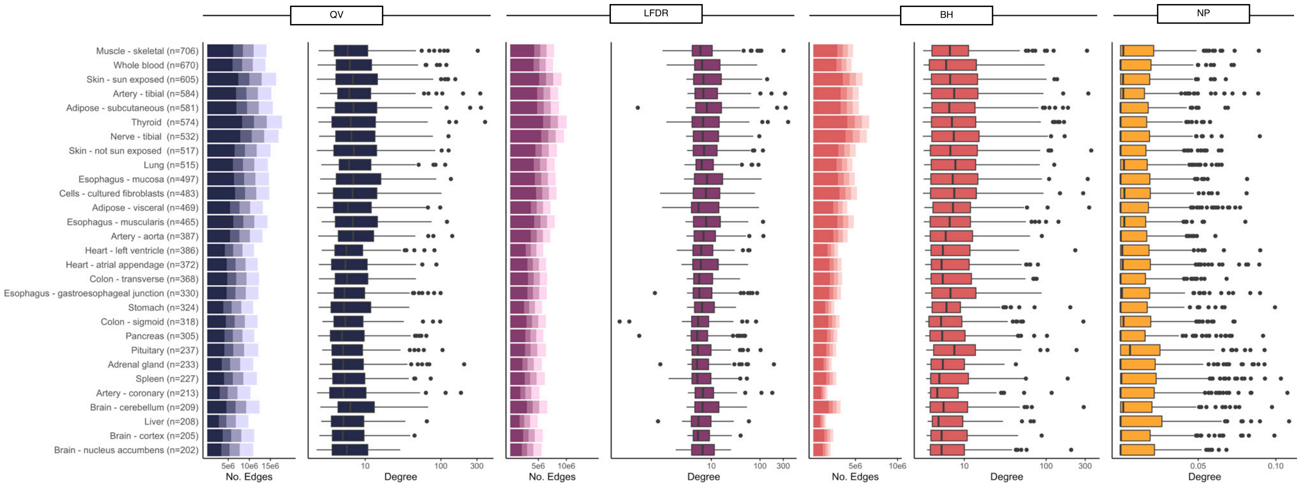

Figure 2. eQTL network edge and SNP degree distributions
The distribution of edges is given by threshold τ from darkest to lightest (0.05, 0.1, 0.15, and 0.2) and definition (q value [QV], local FDR [LFDR], Benjamini-Hochberg [BH], and non-null proportion [NP]); the distribution of degree is given by definition for threshold τ = 0.05 with Z-statistic weights.

attributable to the lower sensitivity when considering eGenes rather than eSNPs (Table S3).

The correlations varied across tissues; the average correlation between the sample splits increases on average with increasing sample size. Previous eQTL studies and power calculations have demonstrated that typically larger sample sizes than these subsamples (n = 101–353) are required to confidently map eQTLs (Huang et al., 2018). There was moderate concordance between the subsample and full-sample degree metrics illustrating the lack of stability of an eQTL network in a small subsample, suggesting the need for a larger minimum sample size for networks and possibly reflecting effects of tissue heterogeneity. The *cis*-specific networks, based on a smaller set of potential eQTLs and thus with a decreased multiple-testing burden, have a notably higher correlation on average than the *trans*-specific networks. The complete networks accounting for location are similar to the *cis*-specific networks, consistent with the dominance of *cis* associations in eQTL analyses. The BH-based SNP degree has a notably higher correlation for the *trans*-specific networks, which may be attributable to the nature of the significance measure calculation, which was estimated per SNP for other measures but considered the complete set of results for the efficient BH computation. The advantage of including both *cis* and *trans* associations is that it allows both local and nonlocal regulatory effects to be modeled and results in networks that exhibit higher-order structure based on those nonlocal associations.

Cross-tissue correlation

We compared the degrees identified in the tissue-specific networks across tissues using Spearman correlation; we expect moderate correlation across all tissues given that *cis*-eQTLs primarily contribute to the degree measures and are more often replicated across tissues (Gamazon et al., 2018), reflecting the fact that cells need to carry out a large number of core processes, such as respiration and metabolism, independent of tissue. This correlation was again performed for the complete, *cis*-, and *trans*-specific networks (Figure 4; Table S4).

Across all eQTLs, the correlation of SNP degree across the tissues was again higher for the BH degree (τ = 0.05; weighted median: 0.27, IQR: 0.14) than the QV-based SNP degree (τ = 0.05; unweighted median: 0.11, IQR: 0.16, weighted median 0.20, IQR: 0.16) and the LFDR-based SNP degree (τ = 0.05; unweighted median: 0.15, IQR: 0.16; weighted median: 0.26, IQR: 0.18). The correlation of NP-based SNP degree across tissues had median -0.33 (IQR: 0.01). This further suggests that this NP-based network method, using a degree based on the estimation of the proportion of non-null hypotheses when it is very small, is not reliable, as we expect positive correlation between tissues for *cis*-specific degrees based on shared genetic regulation.

For all levels of thresholding and for both genome-wide and location-specific definitions, the correlation between tissues is again slightly increased by including weights. Higher, or more relaxed, thresholds led to lower average pairwise correlations between tissues (Table S4). A potential contributor to these trends may be that given the relaxed threshold, there is an increased potential for identifying sub-threshold tissue-specific *trans*-eQTLs as *trans*-eQTLs have lower power for eQTL mapping than *cis*-eQTLs. Given the stability demonstrated by the BH-based network and computational benefits in speed and memory, the subsequent analyses are presented primarily for the BH-based weighted degree with τ = 0.05, and secondarily for all other degrees as reported in the supplemental information.

Relationship to other gene networks

Many types of networks have been used to study biological systems, including correlation-based and regulatory networks. We compared the eQTL networks with gene regulatory and gene-gene correlation networks via the gene degree. We constructed tissue-specific gene regulatory networks using Passing Attributes between Networks for Data Assimilation (PANDA), which uses gene expression, transcriptomic, and transcription factor protein-protein interaction data to infer regulatory associations

CellPress
OPEN ACCESS

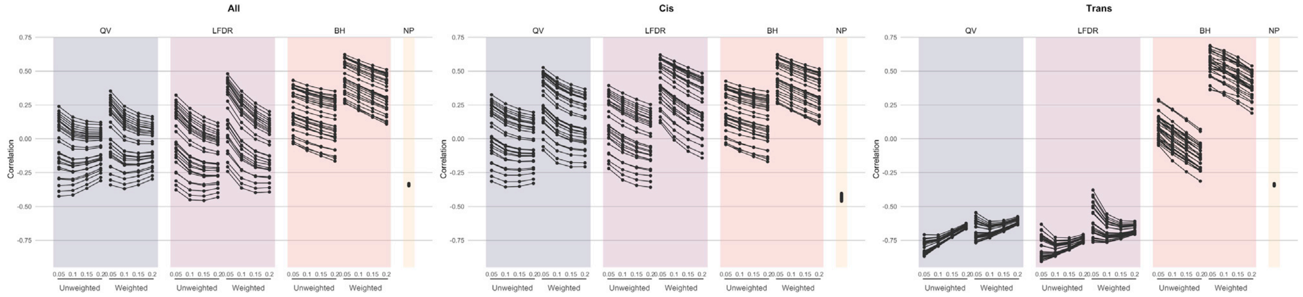

Figure 3. Correlation of SNP degree measures within tissue samples

The correlation of estimated SNP degrees across split tissue samples is given using the unweighted and Z-statistic weighted approaches based upon the QV, LFDR, BH, and NP for each threshold τ (0.05, 0.1, 0.15, and 0.2) given on the X axis. The distribution is given for the full network and stratified into *cis*- and *trans*-location-specific networks.

between transcription factors and target genes (Glass et al., 2013; Schlauch et al., 2020). PANDA network analysis provides insight into genes' regulatory function as targets. We also used weighted correlation network analysis (WGCNA), which builds gene-gene pairwise correlation networks based on gene expression data (Langfelder and Horvath, 2020). We compared the tissue-specific correlation of the gene degree in the eQTL networks to gene degree in PANDA and WGCNA networks in each tissue and then performed meta-analysis (Tables S5 and S6).

The meta-analysis correlation was estimated to be 0.05 (95% confidence interval [CI]: (0.04, 0.07)) between the BH-based weighted gene degree (τ = 0.05) and the degree from the PANDA network. PANDA networks, like eQTL networks, seek to capture genetic regulatory processes, which may explain their positive correlation. The gene eQTL network degree and WGCNA degree had a meta-analysis correlation of -0.02 (95% CI: (-0.04, -0.01)). The correlations were replicated using other sparse network definitions (Table S6). These incongruent findings between other gene network types are consistent with the notion that co-expression and regulatory networks capture different biological features. A correlation-based network finds genes whose expression levels are similar to each other; genes in an eQTL network are expressed at a level correlated with the genotype at that locus. Further, *cis*-eQTLs, which are the dominant edges in the network, generally are associated with SNPs falling in regulatory regions, potentially disrupting transcription-factor binding. Thus the extent of a gene's complex regulatory role would more likely be similarly reflected in both eQTL and gene regulatory networks.

Degree correlates with genetic diversity

We assessed the relationship between the degree and genetic evolution and diversity by calculating the correlation of gene-level degree (BH-based weighted gene degree, τ = 0.05) and both nucleotide diversity and Tajima's D at the gene level (Danecek et al., 2011; Nei and Li, 1979; Tajima, 1989). Nucleotide diversity measures genetic variation based on the number of nucleotide differences between sequences, permitting insight into a population's mutation rate. Tajima's D considers nucleotide differences as well as the number of segregating sites to then assess whether neutral evolution, as in mutation-drift equilibrium, or selection is occurring. The meta-analysis correlation across tissues between gene degree and nucleotide diversity was estimated as 0.13 (95% CI: (0.12, 0.15)); the correlation between gene degree and Tajima's D was estimated as 0.15 (95% CI: (0.14, 0.15)). These results, consistent across sparse degree definitions (Tables S7 and S8), show statistically significant, positive associations between network gene degrees and genetic diversity. Given that eGenes (genes whose expressions are associated with at least one eQTL) are, by definition, more connected in the network, this is consistent with previous findings in plants where eGenes had higher genetic variation and Tajima's D (Mähler et al., 2017). Thus, this similarly suggests that genes less connected within the network are under relatively stronger selective constraint. These results indicate that genes that are more central to the network experience increased rates of molecular evolution, at least in terms of the regulatory processes that control them, and evolve under decreased selective constraint. However, previous analyses have found that eQTL network hubs are less likely to be associated through GWAS with disease processes than are nodes of intermediate degree (Platig et al., 2016; Fagny et al., 2017), indicating this flexibility in regulatory constraint might also be linked to the functional roles played by these eGenes.

Heritability enrichment of degree

We evaluated whether the degree was enriched for trait heritability using S-LDSC on a set of six tissue-specific networks (artery aorta, coronary, and tibial; heart atrial appendage and left ventricle; whole blood) and seven relevant complex blood-related traits (eosinophil, high- and low-density lipoproteins [HDLs and LDLs, respectively], platelet count, red blood cell [RBC] width, red cell count, and white cell count) as analyzed in UK Biobank (Hormozdiari et al., 2018). We considered both SNP- and gene-level annotations (BH-based weighted degree, τ = 0.05). We conditioned, on the baseline-LD model, a heritability model comprised of 97 annotations that has been demonstrated to be highly informative by capturing functionality, conservation, histone marks, and other variant-specific features (Gazal et al., 2017). We thus account for these existing functional annotations and evaluate the added value of our network annotations in capturing trait heritability. We identified the greatest enrichment across networks for the trait RBC width, with estimates ranging from 3.29 ($p = 8.84 \times 10^{-6}$) to

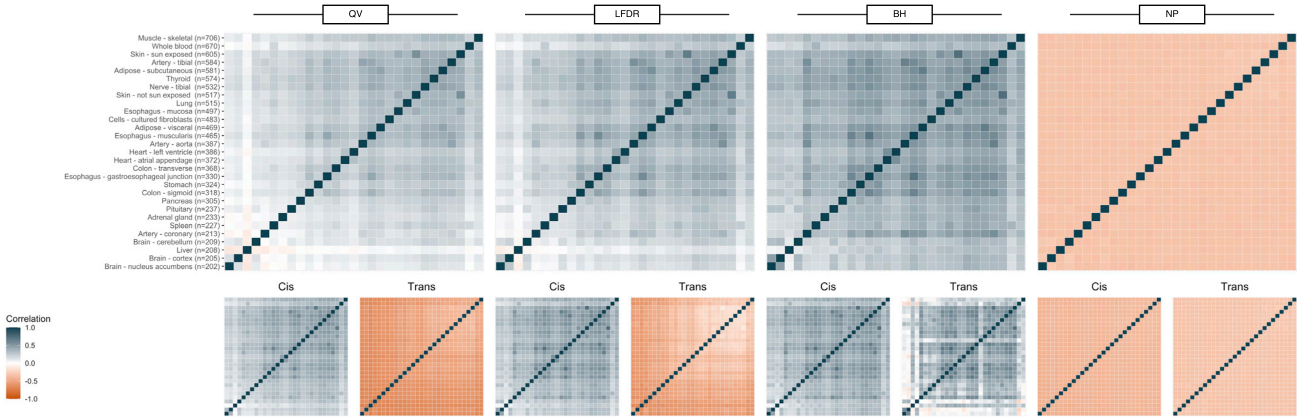

Figure 4. Correlation of SNP degree measures between tissue samples

The pairwise correlations between tissues under the SNP degree definitions for QV, LFDR, BH, and NP, thresholded with $\tau = 0.05$ and weighted by Z-statistics, are given for the complete network and stratified into location-specific networks.

4.25 ($p = 2.06 \times 10^{-9}$) for the SNP-based annotation and ranging from 1.87 ($p = 2.55 \times 10^{-9}$) to 2.45 ($p = 2.25 \times 10^{-11}$) for the gene-based annotation (Figure 5). The degree annotation was significantly enriched for the majority of tissue-trait pairings, for both the SNP- and gene-level degrees (Tables S9, S10, and S11). However, none of the τ^* estimates were significant in these analyses, a measure that accounts for other functional annotations. Thus, given the lack of statistical significant of the effect size τ^*, similar to previously constructed network annotations, the network annotations do not provide significant heritability enrichment beyond the baseline-LD model (Kim et al., 2019).

Computational cost

The network construction and metric calculation methods we used incurred significantly different computational burdens. The least costly method is the BH-based approach. This approach only requires storing SNP-gene associations meeting the particular threshold τ in memory. Further, the degree is calculated via simple summation. Given the high proportion of null associations, this means oftentimes that the association will not need to be stored in memory, and one can capitalize upon current eQTL software that allows for efficient regression for large datasets (Shabalin, 2019). The QV- and LFDR-based approaches require retaining the p values for all associations intermediately, but they can be stored sparsely. The most computationally costly approach is the NP-based network, which requires an edge weight to be calculated and retained in memory for all tested associations. A summary of the computational impact is given in Table S12. We compared the computational impact of calculating the degree of 10,000 SNPs for our considered set of 24,634 genes in the largest tissue (skeletal muscle) repeated across five iterations. The run time, including I/O, was based on a 2.70 GHz laptop with 16 Gb of memory. We observe that the location-specific computation, retaining only edges meeting the minimum threshold for each of *cis* and *trans* edges, is over four times faster than the exhaustive genome-wide computation. This is significant considering that studies are currently growing in the number of genotyped SNPs as well as those that can be imputed with high confidence; therefore, scalability is of high importance.

DISCUSSION

We performed a comprehensive analysis of eQTL networks in twenty-nine different tissues from the GTEx project. Using this unique resource, we tested multiple approaches to reconstructing these networks and interrogated the stability of their resulting properties. We found that the threshold significantly impacted network size, with more stringent thresholds yielding sparser, consistently defined networks. We also observed that the stability of our edge definitions propagated through to affect node degree as well, where correlation-based analyses demonstrated that degree estimates were more consistent in split-sample and cross-tissue networks using these more stringent, sparse definitions. We explored the relationship between eQTL networks and gene regulatory networks, observing a positive correlation between gene degree in our constructed eQTL networks and gene regulatory networks. There was a negative correlation between gene degree in our eQTL networks and gene co-expression networks, suggesting that eQTL networks may be appropriate to consider alongside other networks, particularly as eQTL networks uniquely permit the inclusion of SNPs as nodes and so sample the genetic factors associated with phenotype. We also observed a connection between eQTL network topology and evolutionary processes. Specifically, more highly connected genes in eQTL networks correlate with increased evolutionary rates, indicating that they are reflective of evolutionary processes. We also observe heritability enrichment of blood-related traits for highly connected SNPs and genes in trait-relevant tissues, indicating the informativeness of these network features.

We found that a thresholding approach for constructing network edges was both highly computational efficient and led to stable network properties, including degree. In particular, a weighted BH-based network had the highest correlations in split samples and across tissues; the other thresholding approaches

CellPress
OPEN ACCESS

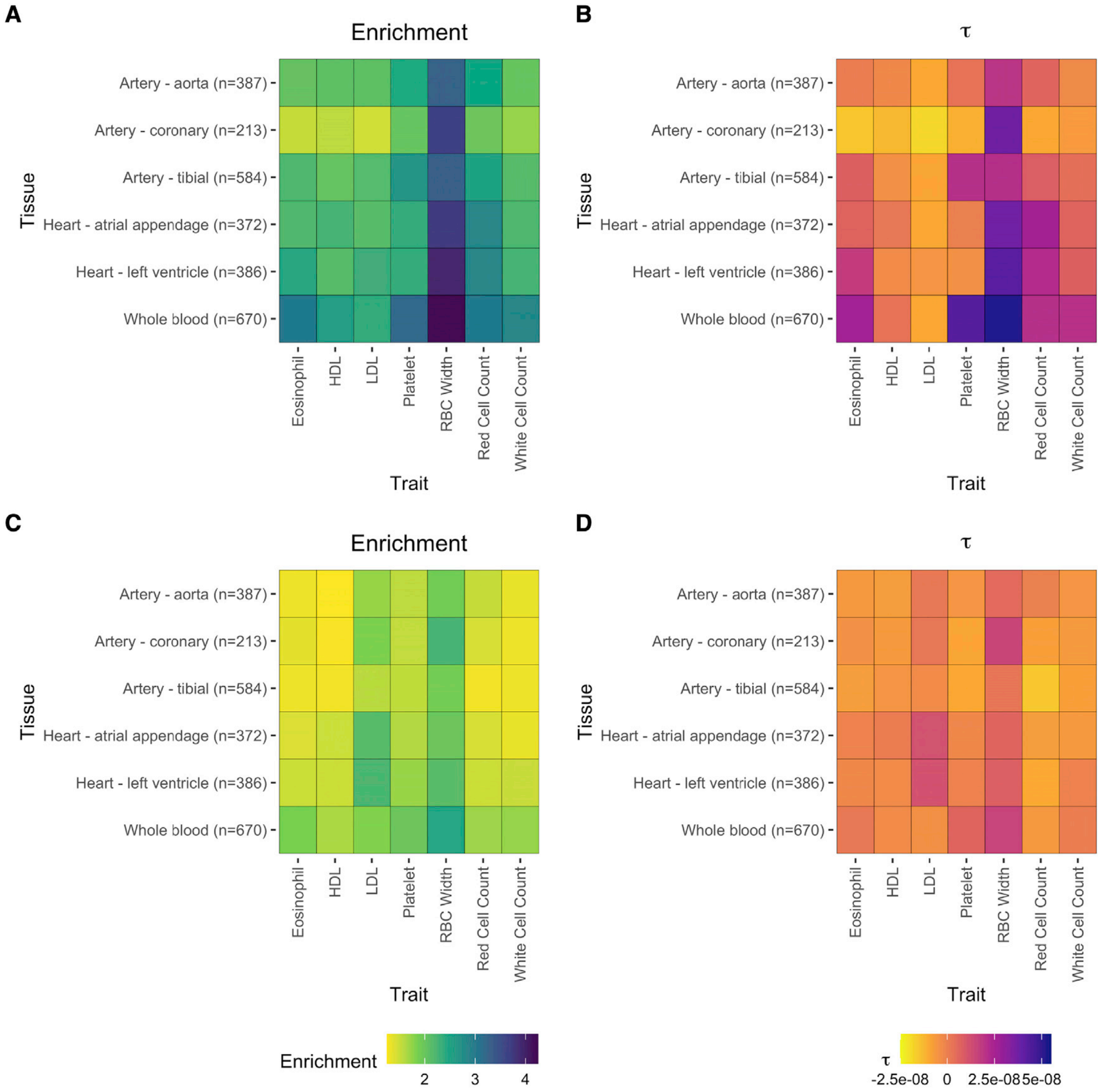

Figure 5. Heritability enrichment and τ* estimates for degree conditional on the baseline-LD model

(A–D) The estimates for SNP-level degree are given in (A) and (B), and estimates for gene-level degree are given in (C) and (D) across tissues and traits. SNPs or genes are included in the annotation given they are in the upper quartile of degree metrics for the BH degree, thresholded with τ = 0.05 and weighted by the Z-statistic.

often performed well with similar trends. We also observed this consistency of the thresholding approach when we compared the eQTL network metrics with gene networks and heritability measures. Further, the thresholded methods straightforwardly separate between *cis*- and *trans*-eQTLs when calculating the FDR rates in all settings (since they are based on location-specific and genome-wide associations, respectively) and likely reflects the fact that *trans* effects are biologically less common than *cis* effects.

The NP-based method did not perform well, which may be because the null proportion is challenging to stably estimate. This measure of the proportion of signals seeks to account for sparsity, and given that eQTL signals are very sparse among a large number of tests, methods for estimating the proportion of non-null do not work well (Storey et al., 2003; Storey, 2002). Furthermore, this approach does not capture the distinction between *cis* and *trans* effects and considers all eQTLs together in genome-wide analysis; as a continuous measure, this may be too stringent since the *cis* and *trans* effects and mechanisms are different.

Our degree findings are also of computational importance given the substantial differences in the computational cost of inferring network edge weights. The BH-, QV-, and LFDR-based networks require fewer computational resources as they can be computed using matrix computations from summarized eQTL mapping results; the use of more stringent thresholds further reduce network-storage requirements and is supported by the consistent results on the downstream analyses across

thresholds. The NP degree measure, similar to many other existing network approaches, requires one to exhaustively output all eQTL relationships and then perform estimation on the entirety of the output. All of these degree measures can be completely parallelized for optimal computation, but the impact is nonetheless an important consideration, as one would expect to periodically repeat such analyses genome wide as datasets evolve.

We have been able to characterize the degree of nodes in eQTL networks under different settings and explore their biological implications. Further methodological work would include pursuing fully weighted representations of the eQTL network while calculating an estimate of the proportion of null for an SNP stratified by *cis*- and *trans*-eQTLs. It would be interesting to apply this framework to other biological QTL networks and so to allow for comparisons across QTL networks, as one would expect that SNPs associated with particular traits would also affect the expression of genes encoding proteins that regulate relevant biological processes. The value of considering network metrics is demonstrated in the secondary analyses, where their distinction from other networks and relationship to genomic features such as diversity are shown. These results further support integrating eQTLs with other measures of genetic association with phenotype.

The results we present here were based on eQTL networks, where the edge weights linking SNPs and genes are based on analysis of experimental data to identify associations between the genotype at a locus and the expression of each gene in the genome. However, the lessons we learned are broadly applicable to a wide range of problems in the inference of biological networks. Many real-world network analyses focus on metrics such as degree or betweenness centrality after binarizing the edges. However, in analyzing biological networks, we often have imperfect evidence or are modeling processes that are neither always ''on'' or ''off'' but instead occur with some likelihood. Understanding how edges are estimated, and the effect that different methodological choices have on downstream analyses and the overall stability of results, is important for further optimizing network methods. Robust methods for network inference and analysis will further our understanding of gene function and help identify downstream relationships with traits and diseases.

Limitations of the study

There are multiple limitations to the work presented here. First, we focused on network specification and its effect on the degree metric using the GTEx V8 data as a test case. While a key metric, the degree may not always be of greatest interest, and thus an association-based bipartite network may be better optimized for a different measure. However, any of the commonly used network metrics are based on edges, and the edge stability analysis we present in the context of degree is likely to affect other measures in a similar manner. We also note that eQTL association analysis methods and software are consistently improving and may permit further optimized computation; the trends we highlight, particularly in terms of storing and accessing large networks, would nonetheless persist. The resulting networks, metrics, and downstream findings may include false positive nodes, particularly in settings with relaxed FDR thresholds. In our analyses, we used the most stringent threshold considered, and trends we observed were largely consistent across the twenty-nine tissues considered. Also, the heritability analysis we performed is limited by previously noted methodological limitations, including that S-LDSC requires sufficiently large and robust annotations for stable estimation, which limited our ability to incorporate weights in this aspect of the analysis.

The challenge that we face in validating network-based methods is that there is no source of genome-wide ''ground truth.'' The work presented here relies on estimating SNP-gene associations through eQTL analysis; these associations are supported by extensive data but are not individually experimentally validated. Consequently, when investigating thresholds on the individual estimates of SNP-gene associations, we must rely on other measures. Here, we appeal to properties such as network stability for identifying optimal methods. This is a reasonable strategy as a growing body of evidence indicates that while individual SNP-gene associations may not be reliable, the overall structure of eQTL networks can inform our understanding of the biology of the system under study. Consequently, consistency in estimating the presence of SNP-gene associations can improve our overall understanding of genetic regulatory processes.

STAR★METHODS

Detailed methods are provided in the online version of this paper and include the following:

- KEY RESOURCES TABLE
- RESOURCE AVAILABILITY
  - Lead contact
  - Materials availability
  - Data and code availability
- EXPERIMENTAL MODEL AND SUBJECT DETAILS
- METHOD DETAILS
  - Bipartite eQTL network construction
  - Degree definition and estimation
  - GTEx data set
  - eQTL mapping
  - Degree correlation between and within tissues
  - Gene network construction and comparison
  - Degree and functional annotations
  - Correlation with genetic diversity annotations
  - Effect size and enrichment estimation

SUPPLEMENTAL INFORMATION

Supplemental information can be found online at https://doi.org/10.1016/j.crmeth.2022.100218.

ACKNOWLEDGMENTS

S.M.G. was supported by the National Science Foundation Graduate Research Fellowship (DGE1144152) and by the National Heart, Lung, and Blood Institute of the National Institutes of Health F31 (HL138832-01). J.Q. and J.P. were supported by a grant from the National Cancer Institute (1R35CA220523). J.P. was supported by a grant from the National Heart, Lung, and Blood Institute (K25HL140186). This work was conducted under dbGaP approved protocol 9112. The Genotype-Tissue Expression (GTEx)

CellPress
OPEN ACCESS

Project was supported by the Common Fund of the Office of the Director of the National Institutes of Health and by NCI, NHGRI, NHLBI, NIDA, NIMH, and NINDS.

AUTHOR CONTRIBUTIONS

S.M.G., M.F., J.P., and J.Q. contributed to the conception of the work. S.M.G., M.F., X.L., J.P., and J.Q. contributed to the study design and method development. S.M.G. contributed to analysis, verified the data, and drafted the manuscript. All authors reviewed the manuscript and approved the submitted work.

DECLARATION OF INTERESTS

The authors declare no competing interests.

INCLUSION AND DIVERSITY

While citing references scientifically relevant for this work, we also actively worked to promote gender balance in our reference list.

Received: September 22, 2021
Revised: February 8, 2022
Accepted: April 25, 2022
Published: May 23, 2022

REFERENCES

Albert, F.W., and Kruglyak, L. (2015). The role of regulatory variation in complex traits and disease. Nat. Rev. Genet. *16*, 197–212. https://doi.org/10.1038/nrg3891.

Asratian, A.S., Denley, T.M., and Häggkvist, R. (1998). Bipartite Graphs and Their Applications, *volume 131* (Cambridge University Press).

Badsha, M.B., and Fu, A.Q. (2019). Learning causal biological networks with the principle of mendelian randomization. Front. Genet. *10*, 460. https://doi.org/10.3389/fgene.2019.00460.

Balduzzi, S., Rücker, G., and Schwarzer, G. (2019). How to perform a meta-analysis with R: a practical tutorial. Evid. Base Ment. Health *22*, 153–160. https://doi.org/10.1136/ebmental-2019-300117.

Barabási, A.L., Gulbahce, N., and Loscalzo, J. (2011). Network medicine: a network-based approach to human disease. Nat. Rev. Genet. *12*, 56–68. https://doi.org/10.1038/nrg2918.

Barber, M.J. (2007). Modularity and community detection in bipartite networks. Phys. Rev. E *76*, 066102. https://doi.org/10.1103/physreve.76.066102.

Benjamini, Y., and Hochberg, Y. (1995). Controlling the false discovery rate: a practical and powerful approach to multiple testing. J. Roy. Stat. Soc. B Stat. Methodol. *57*, 289–300. https://doi.org/10.1111/j.2517-6161.1995.tb02031.x.

Cheung, V.G., Spielman, R.S., Ewens, K.G., Weber, T.M., Morley, M., and Burdick, J.T. (2005). Mapping determinants of human gene expression by regional and genome-wide association. Nature *437*, 1365–1369. https://doi.org/10.1038/nature04244.

Cho, M.H., McDonald, M.L.N., Zhou, X., Mattheisen, M., Castaldi, P.J., Hersh, C.P., DeMeo, D.L., Sylvia, J.S., Ziniti, J., Laird, N.M., et al. (2014). Risk loci for chronic obstructive pulmonary disease: a genome-wide association study and meta-analysis. Lancet Respir. Med. *2*, 214–225. https://doi.org/10.1016/S2213-2600(14)70002-5.

Danecek, P., Auton, A., Abecasis, G., Albers, C.A., Banks, E., DePristo, M.A., Handsaker, R.E., Lunter, G., Marth, G.T., Sherry, S.T., et al. (2011). The variant call format and vcftools. Bioinformatics *27*, 2156–2158. https://doi.org/10.1093/bioinformatics/btr330.

DeLuca, D.S., Levin, J.Z., Sivachenko, A., Fennell, T., Nazaire, M.D., Williams, C., Reich, M., Winckler, W., and Getz, G. (2012). Rna-seqc: Rna-seq metrics for quality control and process optimization. Bioinformatics *28*, 1530–1532. https://doi.org/10.1093/bioinformatics/bts196.

Dermitzakis, E.T. (2008). From gene expression to disease risk. Nat. Genet. *40*, 492–493. https://doi.org/10.1038/ng0508-492.

Efron, B., Tibshirani, R., Storey, J.D., and Tusher, V. (2001). Empirical bayes analysis of a microarray experiment. J. Am. Stat. Assoc. *96*, 1151–1160. https://doi.org/10.1198/016214501753382129.

Fagny, M., Paulson, J.N., Kuijjer, M.L., Sonawane, A.R., Chen, C.Y., Lopes-Ramos, C.M., Glass, K., Quackenbush, J., and Platig, J. (2017). Exploring regulation in tissues with eqtl networks. Proc. Natl. Acad. Sci. U S A *114*, E7841–E7850. https://doi.org/10.1073/pnas.1707375114.

Finucane, H.K., Bulik-Sullivan, B., Gusev, A., Trynka, G., Reshef, Y., Loh, P.R., Anttila, V., Xu, H., Zang, C., Farh, K., et al. (2015). Partitioning heritability by functional annotation using genome-wide association summary statistics. Nat. Genet. *47*, 1228–1235. https://doi.org/10.1038/ng.3404.

Gamazon, E.R., Segrè, A.V., Van De Bunt, M., Wen, X., Xi, H.S., Hormozdiari, F., Ongen, H., Konkashbaev, A., Derks, E.M., Aguet, F., et al. (2018). Using an atlas of gene regulation across 44 human tissues to inform complex disease- and trait-associated variation. Nat. Genet. *50*, 956–967. https://doi.org/10.1038/s41588-018-0154-4.

Gazal, S., Finucane, H.K., Furlotte, N.A., Loh, P.R., Palamara, P.F., Liu, X., Schoech, A., Bulik-Sullivan, B., Neale, B.M., Gusev, A., et al. (2017). Linkage disequilibrium–dependent architecture of human complex traits shows action of negative selection. Nat. Genet. *49*, 1421–1427. https://doi.org/10.1038/ng.3954.

Genomes Project Consortium (2015). A global reference for human genetic variation. Nature *526*, 68–74. https://doi.org/10.1038/nature15393.

Glass, K., Huttenhower, C., Quackenbush, J., and Yuan, G.C. (2013). Passing messages between biological networks to refine predicted interactions. PLoS One *8*, e64832. https://doi.org/10.1371/journal.pone.0064832.

Glass, K., Quackenbush, J., Silverman, E.K., Celli, B., Rennard, S.I., Yuan, G.C., and DeMeo, D.L. (2014). Sexually-dimorphic targeting of functionally-related genes in copd. BMC Syst. Biol. *8*, 118. https://doi.org/10.1186/s12918-014-0118-y.

GTEx Consortium (2015). The genotype-tissue expression (gtex) pilot analysis: multitissue gene regulation in humans. Science *348*, 648–660.

Hawkins, R.D., Hon, G.C., and Ren, B. (2010). Next-generation genomics: an integrative approach. Nat. Rev. Genet. *11*, 476–486. https://doi.org/10.1038/nrg2795.

Hormozdiari, F., Gazal, S., Van De Geijn, B., Finucane, H.K., Ju, C.J.T., Loh, P.R., Schoech, A., Reshef, Y., Liu, X., O'connor, L., et al. (2018). Leveraging molecular quantitative trait loci to understand the genetic architecture of diseases and complex traits. Nat. Genet. *50*, 1041–1047. https://doi.org/10.1038/s41588-018-0148-2.

Howey, R., Shin, S.Y., Relton, C., Davey Smith, G., and Cordell, H.J. (2020). Bayesian network analysis incorporating genetic anchors complements conventional mendelian randomization approaches for exploratory analysis of causal relationships in complex data. PLoS Genet. *16*, e1008198. https://doi.org/10.1371/journal.pgen.1008198.

Howey, R., Clark, A.D., Naamane, N., Reynard, L.N., Pratt, A.G., and Cordell, H.J. (2021). A bayesian network approach incorporating imputation of missing data enables exploratory analysis of complex causal biological relationships. PLoS Genet. *17*, e1009811. https://doi.org/10.1371/journal.pgen.1009811.

Huang, Q.Q., Ritchie, S.C., Brozynska, M., and Inouye, M. (2018). Power, false discovery rate and winner's curse in eqtl studies. Nucleic Acids Res. *46*, e133. https://doi.org/10.1093/nar/gky780.

Kendziorski, C.M., Chen, M., Yuan, M., Lan, H., and Attie, A.D. (2006). Statistical methods for expression quantitative trait loci (eqtl) mapping. Biometrics *62*, 19–27. https://doi.org/10.1111/j.1541-0420.2005.00437.x.

Kim, S.S., Dai, C., Hormozdiari, F., van de Geijn, B., Gazal, S., Park, Y., O'Connor, L., Amariuta, T., Loh, P.R., Finucane, H., et al. (2019). Genes with high network connectivity are enriched for disease heritability. Am. J. Hum. Genet. *104*, 896–913. https://doi.org/10.1016/j.ajhg.2019.03.020.

Korte, A., and Farlow, A. (2013). The advantages and limitations of trait analysis with gwas: a review. Plant Methods *9*, 29. https://doi.org/10.1186/1746-4811-9-29.

Langfelder, P., and Horvath, S. (2008). Wgcna: an r package for weighted correlation network analysis. BMC Bioinf. *9*, 559. https://doi.org/10.1186/1471-2105-9-559.

Langfelder, P., and Horvath, S. (2020). WGCNA: weighted correlation network analysis. r package version 1.69. http://horvath.genetics.ucla.edu/html/CoexpressionNetwork/Rpackages/WGCNA/.

Loh, P.R., Danecek, P., Palamara, P.F., Fuchsberger, C., A Reshef, Y., K Finucane, H., Schoenherr, S., Forer, L., McCarthy, S., Abecasis, G.R., et al. (2016). Reference-based phasing using the haplotype reference consortium panel. Nat. Genet. *48*, 1443–1448. https://doi.org/10.1038/ng.3679.

Mähler, N., Wang, J., Terebieniec, B.K., Ingvarsson, P.K., Street, N.R., and Hvidsten, T.R. (2017). Gene co-expression network connectivity is an important determinant of selective constraint. PLoS Genet. *13*, e1006402. https://doi.org/10.1371/journal.pgen.1006402.

Morley, M., Molony, C.M., Weber, T.M., Devlin, J.L., Ewens, K.G., Spielman, R.S., and Cheung, V.G. (2004). Genetic analysis of genome-wide variation in human gene expression. Nature *430*, 743–747. https://doi.org/10.1038/nature02797.

Nei, M., and Li, W.H. (1979). Mathematical model for studying genetic variation in terms of restriction endonucleases. Proc. Natl. Acad. Sci. U S A *76*, 5269–5273. https://doi.org/10.1073/pnas.76.10.5269.

Nicolae, D.L., Gamazon, E., Zhang, W., Duan, S., Dolan, M.E., and Cox, N.J. (2010). Trait-associated snps are more likely to be eqtls: annotation to enhance discovery from gwas. PLoS Genet. *6*, e1000888. https://doi.org/10.1371/journal.pgen.1000888.

Platig, J., Castaldi, P.J., DeMeo, D., and Quackenbush, J. (2016). Bipartite community structure of eqtls. PLoS Comput. Biol. *12*, e1005033. https://doi.org/10.1371/journal.pcbi.1005033.

Purcell, S., and Chang, C. (2015). Plink 1.9. www.cog-genomics.org/plink/1.9.

R Core Team (2020). R: A Language and Environment for Statistical Computing (R Foundation for Statistical Computing). https://www.R-project.org/.

Schadt, E.E., Lamb, J., Yang, X., Zhu, J., Edwards, S., GuhaThakurta, D., Sieberts, S.K., Monks, S., Reitman, M., Zhang, C., et al. (2005). An integrative genomics approach to infer causal associations between gene expression and disease. Nat. Genet. *37*, 710–717. https://doi.org/10.1038/ng1589.

Schlauch, D., Paulson, J.N., Young, A., Quackenbush, J., and Glass, K. (2020). PANDA algorithm. r package version 1.20.0. https://git.bioconductor.org/packages/pandaR.

Sedgewick, A.J., Buschur, K., Shi, I., Ramsey, J.D., Raghu, V.K., Manatakis, D.V., Zhang, Y., Bon, J., Chandra, D., Karoleski, C., et al. (2019). Mixed graphical models for integrative causal analysis with application to chronic lung disease diagnosis and prognosis. Bioinformatics *35*, 1204–1212. https://doi.org/10.1093/bioinformatics/bty769.

Shabalin, A.A. (2012). Matrix eqtl: ultra fast eqtl analysis via large matrix operations. Bioinformatics *28*, 1353–1358. https://doi.org/10.1093/bioinformatics/bts163.

Shabalin, A.A. (2019). MatrixEQTL: matrix eQTL: ultra fast eQTL analysis via large matrix operations. r package version 2.3. http://www.bios.unc.edu/research/genomic_software/Matrix_eQTL/.

Sonawane, A.R., Platig, J., Fagny, M., Chen, C.Y., Paulson, J.N., Lopes-Ramos, C.M., DeMeo, D.L., Quackenbush, J., Glass, K., and Kuijjer, M.L. (2017). Understanding tissue-specific gene regulation. Cell Rep. *21*, 1077–1088. https://doi.org/10.1016/j.celrep.2017.10.001.

Storey, J.D. (2002). A direct approach to false discovery rates. J. Roy. Stat. Soc. B Stat. Methodol. *64*, 479–498. https://doi.org/10.1111/1467-9868.00346.

Storey, J.D., and Tibshirani, R. (2003). Statistical significance for genomewide studies. Proc. Natl. Acad. Sci. U S A *100*, 9440–9445. https://doi.org/10.1073/pnas.1530509100.

Storey, J.D. (2003). The positive false discovery rate: a bayesian interpretation and the q-value. Ann. Stat. *31*, 2013–2035. https://doi.org/10.1214/aos/1074290335.

Storey, J.D., Taylor, J.E., and Siegmund, D. (2004). Strong control, conservative point estimation and simultaneous conservative consistency of false discovery rates: a unified approach. J. Roy. Stat. Soc. B Stat. Methodol. *66*, 187–205. https://doi.org/10.1111/j.1467-9868.2004.00439.x.

Storey, J.D., Bass, A.J., Dabney, A., and Robinson, D. (2019). qvalue: Q-value estimation for false discovery rate control. r package version 2.18.0. http://github.com/jdstorey/qvalue.

Szklarczyk, D., Morris, J.H., Cook, H., Kuhn, M., Wyder, S., Simonovic, M., Santos, A., Doncheva, N.T., Roth, A., Bork, P., et al. (2017). The string database in 2017: quality-controlled protein–protein association networks, made broadly accessible. Nucleic Acids Res. *45*, D362–D368. https://doi.org/10.1093/nar/gkw937.

Tajima, F. (1989). Statistical method for testing the neutral mutation hypothesis by dna polymorphism. Genetics *123*, 585–595. https://doi.org/10.1093/genetics/123.3.585.

Wang, K., Li, M., and Hakonarson, H. (2010). Analysing biological pathways in genome-wide association studies. Nat. Rev. Genet. *11*, 843–854. https://doi.org/10.1038/nrg2884.

Ward, L.D., and Kellis, M. (2012). Interpreting noncoding genetic variation in complex traits and human disease. Nat. Biotechnol. *30*, 1095–1106. https://doi.org/10.1038/nbt.2422.

Weirauch, M.T., Yang, A., Albu, M., Cote, A.G., Montenegro-Montero, A., Drewe, P., Najafabadi, H.S., Lambert, S.A., Mann, I., Cook, K., et al. (2014). Determination and inference of eukaryotic transcription factor sequence specificity. Cell *158*, 1431–1443. https://doi.org/10.1016/j.cell.2014.08.009.

Yazdani, A., Yazdani, A., Samiei, A., and Boerwinkle, E. (2016). Generating a robust statistical causal structure over 13 cardiovascular disease risk factors using genomics data. J. Biomed. Inform. *60*, 114–119. https://doi.org/10.1016/j.jbi.2016.01.012.

Zhu, J., Lum, P.Y., Lamb, J., GuhaThakurta, D., Edwards, S.W., Thieringer, R., Berger, J.P., Wu, M.S., Thompson, J., Sachs, A.B., et al. (2004). An integrative genomics approach to the reconstruction of gene networks in segregating populations. Cytogenet. Genome Res. *105*, 363–374. https://doi.org/10.1159/000078209.

Zhu, J., Sova, P., Xu, Q., Dombek, K.M., Xu, E.Y., Vu, H., Tu, Z., Brem, R.B., Bumgarner, R.E., and Schadt, E.E. (2012). Stitching together multiple data dimensions reveals interacting metabolomic and transcriptomic networks that modulate cell regulation. PLoS Biol. *10*, e1001301. https://doi.org/10.1371/journal.pbio.1001301.

STAR★METHODS

KEY RESOURCES TABLE

REAGENT or RESOURCE	SOURCE	IDENTIFIER
Deposited data		
GTEx version 8	dbGaP (https://www.ncbi.nlm.nih.gov/projects/gap/cgi-bin/study.cgi?study_id=phs000424.v8.p2) or GTEx Portal (https://gtexportal.org/home/datasets)	dbGaP: phs000424.v8.p2
Software and algorithms		
Matrix eQTL	http://www.bios.unc.edu/research/genomic_software/Matrix_eQTL/	R package version 2.3
GENCODE	https://www.gencodegenes.org/	GENCODE version 26
PANDA	https://git.bioconductor.org/packages/pandaR	R package version 1.20.0
PLINK	https://www.cog-genomics.org/plink/	PLINK version 1.9
WGCNA	http://horvath.genetics.ucla.edu/html/CoexpressionNetwork/Rpackages/WGCNA/	R package version 1.69
VCFtools	https://vcftools.github.io/index.html	Perl package version 0.1.16
All code used for the analysis presented here.	https://github.com/sheilagaynor/connectivity_eqtl_networks	https://doi.org/10.5281/zenodo.6478155

RESOURCE AVAILABILITY

Lead contact
Further information and requests for resources should be directed to and will be fulfilled by the lead contact, John Quackenbush (johnq@hsph.harvard.edu).

Materials availability
No materials were generated for this study.

Data and code availability
All data used for the analyses described in this manuscript were obtained from: the GTEx Portal on 12/17/19 and dbGaP: phs000424.v8 on 12/17/19.

Code for analyses are publicly available online at: https://doi.org/10.5281/zenodo.6478155.

Any additional information required to reanalyze the data reported in this paper is available from the lead contact upon request.

EXPERIMENTAL MODEL AND SUBJECT DETAILS

No experimental models were generated for this study nor were research subjects enrolled.

METHOD DETAILS

In this section, we describe our approach for constructing eQTL networks and defining the network metric of degree. This approach requires processed genotype and gene expression data, which can then be used to map eQTLs and build a network. We identify differences in the various approaches with regards to stability and computational feasibility. We also provide details of the implementation of these approaches and their reproducibility. An overview of the workflow is given in Figure 1.

Bipartite eQTL network construction
We evaluated expression quantitative trait loci (eQTL) by modeling the association between SNP genotypes and gene expression (Kendziorski et al., 2006; Wang et al., 2010). In particular, consider an $r \times n$ matrix S of SNP genotypes and $r \times m$ matrix G of gene expression, each with r rows representing observations and columns representing n SNPs and m genes, respectively. Consider

a covariate matrix X, including features such as principal components for population structure, sex, and age. We model the eQTL of a particular SNP i on a locus's gene expression j:

$$G_j = \mathbf{X}^{\mathbf{T}}\alpha + \beta_{ij}S_i.$$

Associations are considered for both SNPs acting to influence expression in *cis* or *trans*, where SNPs within 1MB of a gene's transcription start site are considered local or in *cis*.

The eQTL associations between all pairs of SNPs and genes can be represented as a bipartite network by considering each SNP i and gene j to be a node in the network, and casting a function of their association as edges. We define elements $a_{i,j}$ of the $n \times m$ upper right block of the bipartite network adjacency matrix A based on the association of the SNP-gene pairings. Previous studies of eQTL networks took A to be a binary matrix, where matrix elements were defined by dichotomizing all SNP-gene associations according to a fixed cutoff q = 0.2 on the false discovery rate (FDR) of the eQTL regression, $I_{i,j}\{FDR \angle q\}$ (Platig et al., 2016; Fagny et al., 2017). Thus, when the estimated FDR of the eQTL regression was below the threshold of 0.2 for SNP node i and gene expression node j then $a_{i,j} = -1$, indicating there was an edge connecting the nodes, and $a_{i,j} = 0$ otherwise.

We define a set of adjacency matrix representations based on summary statistics from eQTL analyses. We first consider sparse representations of A. Sparsity makes biological sense as even disease-associated SNPs are known to generally have small effect sized, meaning they unlikely to exert their influence across the genome. In this setting, sparsity is enforced by thresholding a summary statistic to determine non-zero edges. Each element of A is defined as $a_{i,j} = |z_{i,j}| I\{Y_{i,j} < \tau\}$, where $Z_{i,j}$ is either set equal to 1 for an unweighted representation or the z-statistic for testing $\beta_{i,j}$ from the eQTL regression between SNP i and gene j for a weighted representation, $Y_{i,j}$ is a measure of the significance of the eQTL association where $\tau \in 0.05, 0.1, 0.15, 0.2$. Therefore when the estimated regression measure was below the threshold of τ for the SNP-gene pairing, then $a_{i,j} = |z_{i,j}|$ and $a_{i,j} = 0$ otherwise, providing a sparse representation incorporating the magnitude of the effect. We estimate the network edges across all associations both without delineation to location and stratified between *cis*- and *trans*-eQTLs.

We consider three definitions of Y, each providing a measure of significance to account for multiple comparisons: q-value (Storey, 2002; Storey and Tibshirani, 2003; Storey et al., 2003), local FDR (Efron et al., 2001; Storey et al., 2003), and an adaptation of Benjamini-Hochberg FDR (Benjamini and Hochberg, 1995). We first consider the q-value, a quantity that controls the FDR by providing the minimum FDR at which an eQTL association test is called significant. The q-value is estimated in practice as $\hat{q} - value(p_i) = min_{t \geq p_i} \widehat{FDR}(t)$ for a given p-value p_i. We next consider the local FDR, which estimates the posterior probability that the null hypothesis of no eQTL association is true, given the value of test statistic. The local FDR is given as $lFDR(z) = \pi_0 f_0(z)/f(z)$ where $f(z) = \pi_0 f_0(z) + \pi_1 f_1(z)$ for the null probability and density $\pi_0, f_0(z)$ and non-null probability and density $\pi_1, f_1(z)$. The q-value and local FDR measures are implemented in the qvalue R package (Storey et al., 2019). We last considered an adaptation of the Benjamini-Hochberg FDR, which is calculated for p-values meeting a significance threshold (and so not considering those non-significant) for computational efficiency (Shabalin, 2012). This approach considers the K significant p-values from N total tests, and calculates FDR as $q_K = \frac{N}{K} p_K, q_i = \min\left(\frac{N}{i} p_i, q_{i+1}\right)$ for $i = 1, \ldots, K - 1$. This procedure is provided in the MatrixEQTL R package (Shabalin, 2019).

We also consider a denser representation of A, where $a_{i,j} = p_{ij}$ where $p_{i,j}$ is the nominal p-value for the Z-test of the eQTL regression parameter $\beta_{i,j}$ between SNP i and gene j. In contrast to the biological assumptions leading to a sparsity requirement, a denser network allows us to capture the fact that we have no prior knowledge of precisely which SNPs and genes might have an association and so allows us to estimate the weight of evidence supporting an interaction. As such, this representation includes the p-value of all eQTL associations and does not involve thresholding. These sparse and denser adjacency matrix representations are thus defined as the following, with n rows of SNPs and m columns of genes:

$$\mathbf{B}_{sparse} = \begin{bmatrix} |z_{1,1}| I_{1,1}\{Y < \tau\} & \cdots & |z_{1,m}| I_{1,m}\{Y < \tau\} \\ |z_{2,1}| I_{2,1}\{Y < \tau\} & \cdots & |z_{2,m}| I_{2,m}\{Y < \tau\} \\ \vdots & \ddots & \vdots \\ |z_{n,1}| I_{n,1}\{Y < \tau\} & \cdots & |z_{n,m}| I_{n,m}\{Y < \tau\} \end{bmatrix}, \mathbf{B}_{denser} = \begin{bmatrix} p_{11} & \cdots & p_{1m} \\ p_{21} & \cdots & p_{2m} \\ \vdots & \ddots & \vdots \\ p_{n1} & \cdots & p_{nm} \end{bmatrix}$$

Degree definition and estimation

To identify nodes (either SNPs or genes in our bipartite representation) that are central to the network, we consider the network metric of degree. For an eQTL network, a SNP with high degree is most highly connected to the expression of genes and therefore should be highly functionally relevant. We define the node-level metric of degree particular to each adjacency matrix definition. For the sparse representation of A, the degree of SNP i and the degree of gene j are defined as follows:

$$d^{SNP}_{sparse} = \sum_{j=1}^{m} |z_{i,j}| I_{i,j}, d^{Gene}_{sparse} = \sum_{i=1}^{n} |Z_{i,j}| I_{i,j}.$$

In the sparse, unweighted adjacency setting, all edges are binary. Thus, we take the standard summation of binary edges to obtain a count of the number of edges or connections a SNP has to a gene (or a gene to all SNPs). For the sparse weighted setting, the degree incorporates the magnitude of the test statistic $Z_{i,j}$ as a weighted sum.

For the denser representation of A, we estimate the proportion of significant eQTL analyses for a particular SNP, or the proportion of genes whose expression are influenced by the SNP by utilizing the proportion of true null hypotheses. The proportion of null hypotheses is given as $\rho_0 = m_0/m$ where m_0 is the number of true null hypotheses and m is the total number of hypotheses. (Storey, 2002; Storey et al., 2003, 2004; Storey and Tibshirani, 2003). As such, the degree is given as:

$$d^{SNP}_{denser} = 1 - \rho_0(p_{i,1}, p_{i,2}, \ldots, p_{i,m}); d^{Gene}_{denser} = 1 - \rho_0(p_{1,j}, p_{2,j}, \ldots, p_{n,j})$$

Thus SNPs that have higher degree if they are estimated to have fewer true null associations to the genes. This degree d_{NP}, thus is given by the estimation of the proportion of non-null hypotheses.

GTEx data set

We downloaded data from the NHGRI Genotype-Tissue Expression (GTEx) project to build eQTL networks in each of twenty-nine tissues. The GTEx project is a consortium collecting genotype and expression data from multiple human tissues from hundreds of human donors. We downloaded the Version 8.0 whole genome sequencing and RNA-seq data from the database of Genotypes and Phenotypes (dbGaP): phs000424.v8.p2. A threshold of at least 200 individuals per tissue available was considered for appropriate statistical power and network stability; sex-specific tissues were not included. Computations on the GTEx data were run on the Bridges system at the Pittsburgh Supercomputing Center (PSC) and the Cannon cluster supported by the Faculty of Arts and Sciences Division of Science, Research Computing Group at Harvard University. The sequencing data were processed in Plink 1.90 to retain only SNPs, and remove variants with genotype missingness greater than 10% or minor allele frequency less than 0.05 (Purcell and Chang, 2015); SNP imputation was performed using Eagle2 (Loh et al., 2016). Fully processed, filtered and normalized RNA-Seq data were obtained from the GTEx Portal (www.gtexportal.org). Briefly, the GENCODE 26 model was used to collapse transcripts and quantify using RNA-SeQC (DeLuca et al., 2012).

eQTL mapping

We used a linear regression model with covariates assuming an additive effect of genotypes to map eQTLs. We accounted for population stratification by using the first five principal components of the genotypes as covariates. We further adjusted for sex, genotyping platform and protocol, and the GTEx-recommended set of f PEER factors based on sample size, with the model for gene j and SNP i given by $G_j = \beta_{ij}S_i + \alpha_0 + \alpha_1 PC_1 + \ldots + \alpha_5 PC_5 + \alpha_4 Sex + \alpha_5 PCR + \alpha_6 Platform + \alpha_7 PEER_1 + \ldots \alpha_{7+f-1} PEER_f$. Functions of the regression coefficient β_{ij} were then used in network construction as previously described. Wald tests were used for performing inference on β_{ij}, and nominal p-values were considered throughout.

We defined *cis*-eQTLs to be SNPs within 1MB of a gene's transcription start; all other SNP-gene pairings were defined as *trans*-eQTLs. Analyses were conducted in R 3.3.0 and utilized the aforementioned MatrixEQTL and qvalue packages (R Core Team, 2020). All calculations were massively parallelized across SNPs (Shabalin, 2012). The eQTL mapping by the GTEx Consortium was compared by downloading the single-tissue *cis*-eQTL results for significant variant-gene associations based on permutations from the GTEx Portal.

Degree correlation between and within tissues

We calculated the correlation of a given degree metric in two settings: across different tissues and within a particular tissue. We compared the degree of SNPs and genes between tissues via correlation to define the network-level relationship between tissues. We expected, particularly for *cis*-eQTLs, that tissue-specific networks would share features. Given the non-normal distributions of each of the degree measures, we used Spearman correlation. We further considered the correlation of the degree within a particular tissue, predominantly as a demonstration of reproducibility for each degree measure. We randomly split the observations for each tissue into two equal sets, constructed networks and calculated the defined degree, and then estimated the correlation of the degree between the splits. This was repeated five times for each tissue to account for variability. Lastly, we considered the correlation within a particular tissue for different degree metrics to evaluate the impact of weighting on correlation.

Gene network construction and comparison

We built tissue-specific gene expression correlation networks to compare network-based relationships between genes. We used Weighted Gene Co-expression Network Analysis, as implemented in the WGCNA R package, to construct a network defined by the correlation pattern of genes across the GTEx expression data (Langfelder and Horvath, 2008, 2020). This approach requires the selection of a soft thresholding power for constructing the network, which was selected based on inspecting by plot the first inflection point for the scale-free topology fit index curve. The co-expression network was constructed using all of the genes considered in eQTL mapping; the degree was calculated using the *intramodularConnectivity* function to obtain the total connectivity.

We also built tissue-specific regulatory networks using PANDA as described by Sonawane et al. (2017). In particular, we used the pandaR package in Bioconductor (Glass et al., 2013; Schlauch et al., 2020) with a prior network provided by mapping transcription

factor binding motifs to the genome, protein-protein interaction data derived from the Catalog of Inferred Sequence Binding Preferences and StringDb (Weirauch et al., 2014; Szklarczyk et al., 2017), and gene expression data from GTEx V8 as inputs to PANDA. The output from PANDA was a set of twenty-nine tissue-specific gene regulatory network models linking transcription factors to genes for which there is evidence of regulation. We calculated the degree of genes in the regulatory network using the following proposed transformation to the edge weights to account for negative edge weights,

$$W_{ij} = \ln\left(e^{w_{ij}} + 1\right),$$

where w_{ij} is the edge weight between transcription factor i and gene j.

We compared the degree across the different types of gene networks, exploring the differences in the regulatory relationships represented in these networks. We calculated the correlation of the gene degree between our proposed eQTL network, primarily considering the Benjamini-Hochberg based weighted gene degree with a threshold of $\tau < 0.05$ as well as the other sparse definitions within the supplemental information, and each of the co-expression and regulatory networks for each tissue. We then meta-analyzed the correlations using a random effects model using the meta package (Balduzzi et al., 2019).

Degree and functional annotations

We define genomic annotations based on the estimated SNP and gene degrees. For the SNP-level degree, we defined the annotation to be the Benjamini-Hochberg based weighted degree with a corresponding threshold of $\tau < 0.05$. For the gene-level degree, we annotated all SNPs within the gene ($\pm$ 50 KB window) with the gene's continuous-valued Benjamini-Hochberg based weighted degree with a corresponding threshold of $\tau < 0.05$. We also defined annotations for LFDR and QV with a threshold of $\tau < 0.05$. We used the 1000G European samples as reference SNPs for defining the gene-based annotation (Genomes Project Consortium, 2015).

We also considered two sets of external annotations. First, we estimate annotations capturing genetic diversity. We calculate both nucleotide diversity (π) and Tajima's D across all genes for which expression was measured. We used the *window-pi* and *TajimaD* functions of VCFtools on the previously described GTEx genotype data (Danecek et al., 2011). Second, we use the baseline-LD model (v2.2) in enrichment estimation, which contains a broad set of 97 annotations (Gazal et al., 2017). This model extends previous baseline-LD models and captures variant characteristics including functional regions, conservation, MAF, and LD-related annotations.

Correlation with genetic diversity annotations

We evaluated the correlation between degree and genetic diversity. For each tissue, we used the gene-level degree defined above primarily Benjamini-Hochberg based weighted degree with a corresponding threshold of $\tau < 0.05$, and secondarily across the other sparse definitions) and calculated the correlation to nucleotide diversity and Tajima's D in order to assess whether there is a relationship between increased network connections and genetic evolution. We summarized across tissues by meta-analyzing the correlations using a random effects model via the meta package (Balduzzi et al., 2019).

Effect size and enrichment estimation

We used stratified LD score regression (S-LDSC) to estimate the enrichment and standardized effect size of the degree-defined annotations (Gazal et al., 2017; Finucane et al., 2015). In particular, we considered seven blood traits selected to correspond with six relevant tissue-specific networks. The summary statistics for these traits were obtained from a publicly available analysis of the UK Biobank (Hormozdiari et al., 2018). As previously described, we define a_{cj} as the annotation value of SNP j for the annotation c and τ_c as the contribution of annotation c to per-SNP heritability contribution. We consider a binary annotation in order to have sufficiently stable estimates, defined as 1 where the variant is in the top quartile of the Benjamini-Hochberg (or secondarily LFDR or QV) based weighted degree with a corresponding threshold of $\tau < 0.05$ and 0 otherwise. Then, assuming that the variance of each SNP is a linear additive contribution to the annotation:

$$Var(\beta_j) = \sum_c a_{cj}\tau_c,$$

where τ_c is estimated as:

$$E\left[\chi_j^2\right] = N\sum_c l(j,c)\tau_c + 1,$$

where N is the GWAS sample size and $l(j,c)$ is the LD score of SNP j for the annotation c. The LD score is estimated as $l(j,c) = \sum_k a_{ck} r_{jk}^2$ where r_{jk} is the correlation between SNPS j and k. We used LD scores computed from 1000G data from individuals with European ancestry (Genomes Project Consortium, 2015).

The first measure of interest, effect size, is a standardized measure that describes effects unique to annotation c, conditional on all other annotations. It is defined as the proportionate change in per-SNP heritability associated with a one standard deviation increase in the value of the annotation (conditional on all the other annotations in the model),

$$\tau_c^* = \frac{\tau_c sd(c)}{h_g^2 / M},$$

where $sd(C)$ is the standard deviation of annotation c, h_g^2 is the SNP heritability, and M is the number of SNPs used in heritability estimation. p-values were computed assuming $\tau^*/sd(\tau^*) \sim N(0,1)$..

The second measure of interest, enrichment, is the proportion of heritability explained by the annotation divided by the proportion of SNPs in the annotation. It describes effects that are both unique and non-unique. Thus for a continuous annotation, enrichment is given as:

$$Enrichment : \frac{\%h_g^2(C)}{\%SNP(C)} = \frac{h_g^2(C)}{h_g^2} \bigg/ \frac{\sum_j a_{jc}}{M},$$

where $h_g^2(C)$ is the heritability of annotation c. p-values were computed using a block-jacknife.

Cell Reports Methods, Volume *2*

Supplemental information

Connectivity in eQTL networks dictates reproducibility and genomic properties

Sheila M. Gaynor, Maud Fagny, Xihong Lin, John Platig, and John Quackenbush

Table S 1. eQTL findings using the GTEx Version 8.0 whole genome sequencing and RNA-seq data from the present analysis using MatrixEQTL and the GTEx consortium results. Related to STAR methods and Figure 1

Tissue	Samples	*cis*-eQTLs (Present)	*trans*-eQTLs (Present)	*cis*-eQTLs (GTEx)	*cis*-eQTLs (Shared)	Proportion
Adipose - subcutaneous	581	3153343	82228	2930370	2247923	76.7%
Adipose - visceral	469	2305141	62817	2031091	1592478	78.4%
Adrenal gland	233	1182630	23445	959376	784325	81.8%
Artery - aorta	387	2337275	44445	2038599	1605742	78.8%
Artery - coronary	213	910941	19428	721637	593406	82.2%
Artery - tibial	584	3180675	72189	2958254	2258434	76.3%
Brain - cerebellum	209	1807794	28546	1505827	1221888	81.1%
Brain - cortex	205	1286185	24635	1062868	866891	81.6%
Brain - nucleus accumbens	202	1108374	21265	908866	746800	82.2%
Cells - cultured fibroblasts	483	3018543	81912	2890486	2195677	76.0%
Colon - sigmoid	318	1747716	33670	1474957	1182458	80.2%
Colon - transverse	368	1925725	47708	1672709	1321602	79.0%
Esophagus - gastroesophageal junction	330	1863862	37980	1556679	1238733	79.6%
Esophagus - mucosa	497	2850186	74432	2616828	2028925	77.5%
Esophagus - muscularis	465	2782602	62711	2529273	1958724	77.4%
Heart - atrial appendage	372	1887335	33613	1620487	1282994	79.2%
Heart - left ventricle	386	1654386	34254	1409424	1121382	79.6%
Liver	208	806182	18037	629559	523509	83.2%
Lung	515	2684713	69789	2392204	1872011	78.3%
Muscle Skeletal	706	2781020	67568	2601575	2004324	77.0%
Nerve - tibial	532	3738663	86377	3460273	2648324	76.5%
Pancreas	305	1605071	29111	1340448	1069491	79.8%
Pituitary	237	1505724	30499	1226883	999098	81.4%
Skin - not sun exposed	517	2898044	80477	2697337	2090964	77.5%
Skin - sun exposed	605	3406583	97946	3244342	2488165	76.7%
Spleen	227	1503880	30258	1262024	1019990	80.8%
Stomach	324	1387641	29666	1156158	929380	80.4%
Thyroid	574	3930834	110952	3712240	2832547	76.3%
Whole blood	670	2660893	83035	2414653	1860924	77.1%

Table S 2. Correlation of weighted and unweighted degree measures for the sparse degrees defined by Q-value (QV), local FDR (LFDR), and Benjamini-Hochberg (BH). The correlation is provided for both SNP and gene degree across all values of τ considered, where the weight in the weighted network is defined by the Z-statistic. Related to STAR methods and Figure 2

	SNP			Gene		
τ	QV	LFDR	BH	QV	LFDR	BH
0.05	0.87 (0.021)	0.864 (0.018)	0.868 (0.015)	0.863 (0.02)	0.866 (0.018)	0.857 (0.017)
0.1	0.866 (0.019)	0.855 (0.016)	0.868 (0.015)	0.86 (0.019)	0.862 (0.017)	0.85 (0.016)
0.2	0.863 (0.016)	0.844 (0.015)	0.867 (0.016)	0.855 (0.018)	0.854 (0.016)	0.836 (0.017)
0.25	0.861 (0.015)	0.84 (0.024)	0.866 (0.023)	0.853 (0.018)	0.85 (0.016)	0.827 (0.018)

Table S 3. Average correlation of degrees within tissues for all, cis, and $trans$ edges. For each tissue, we calculated the SNP and gene degree for all nodes in split-sample networks, calculated the correlation between splits, and averaged across five iterations. We report the average across all tissues. Related to Figure 3

		SNP			Gene		
	τ	QV	LFDR	BH	QV	LFDR	BH
Unweighted all	0.05	-0.057 (0.195)	0.014 (0.209)	0.214 (0.148)	0.497 (0.106)	0.454 (0.124)	0.389 (0.171)
	0.1	-0.104 (0.168)	-0.082 (0.2)	0.176 (0.15)	0.51 (0.095)	0.475 (0.112)	0.402 (0.189)
	0.15	-0.102 (0.144)	-0.126 (0.18)	0.145 (0.153)	0.519 (0.087)	0.479 (0.107)	0.432 (0.177)
	0.2	-0.082 (0.124)	-0.141 (0.159)	0.12 (0.157)	0.525 (0.082)	0.48 (0.103)	0.457 (0.158)
	0.25	-0.056 (0.109)	-0.138 (0.139)	0.104 (0.162)	0.527 (0.079)	0.479 (0.099)	0.475 (0.138)
Weighted all	0.05	0.047 (0.208)	0.18 (0.222)	0.467 (0.118)	0.478 (0.118)	0.432 (0.133)	0.402 (0.172)
	0.1	-0.039 (0.181)	0.037 (0.218)	0.419 (0.121)	0.48 (0.112)	0.447 (0.123)	0.414 (0.192)
	0.15	-0.06 (0.155)	-0.038 (0.199)	0.375 (0.124)	0.479 (0.107)	0.445 (0.119)	0.445 (0.182)
	0.2	-0.056 (0.135)	-0.075 (0.177)	0.336 (0.128)	0.476 (0.104)	0.441 (0.117)	0.471 (0.164)
	0.25	-0.041 (0.117)	-0.089 (0.156)	0.302 (0.134)	0.47 (0.102)	0.436 (0.113)	0.491 (0.146)
Unweighted cis	0.05	0.052 (0.188)	0.127 (0.184)	0.208 (0.149)	0.517 (0.104)	0.432 (0.151)	0.384 (0.172)
	0.1	-0.008 (0.18)	0.06 (0.189)	0.171 (0.151)	0.555 (0.077)	0.497 (0.12)	0.395 (0.189)
	0.15	-0.032 (0.166)	0.014 (0.185)	0.141 (0.154)	0.565 (0.067)	0.534 (0.098)	0.425 (0.178)
	0.2	-0.039 (0.153)	-0.016 (0.176)	0.117 (0.158)	0.568 (0.062)	0.544 (0.088)	0.45 (0.157)
	0.25	-0.036 (0.141)	-0.035 (0.166)	0.102 (0.163)	0.571 (0.058)	0.544 (0.083)	0.467 (0.137)
Weighted cis	0.05	0.279 (0.185)	0.418 (0.157)	0.465 (0.119)	0.526 (0.113)	0.425 (0.163)	0.396 (0.173)
	0.1	0.189 (0.189)	0.341 (0.178)	0.419 (0.122)	0.566 (0.087)	0.492 (0.13)	0.408 (0.192)
	0.15	0.142 (0.181)	0.277 (0.187)	0.376 (0.125)	0.577 (0.079)	0.531 (0.108)	0.439 (0.182)
	0.2	0.118 (0.171)	0.228 (0.188)	0.338 (0.129)	0.582 (0.074)	0.544 (0.098)	0.465 (0.164)
	0.25	0.11 (0.161)	0.192 (0.184)	0.305 (0.135)	0.587 (0.071)	0.546 (0.094)	0.485 (0.145)
Unweighted $trans$	0.05	-0.813 (0.048)	-0.806 (0.083)	0.086 (0.087)	0.023 (0.008)	-0.057 (0.018)	0.059 (0.097)
	0.1	-0.767 (0.025)	-0.822 (0.043)	0.007 (0.094)	0.029 (0.006)	0.027 (0.007)	-0.249 (0.078)
	0.15	-0.709 (0.016)	-0.792 (0.026)	-0.064 (0.094)	0.035 (0.007)	0.033 (0.006)	-0.403 (0.052)
	0.2	-0.651 (0.012)	-0.748 (0.017)	-0.134 (0.091)	0.046 (0.015)	0.041 (0.011)	-0.49 (0.029)
	0.25	-0.595 (0.01)	-0.697 (0.012)	-0.2 (0.087)	0.061 (0.027)	0.054 (0.021)	-0.539 (0.016)
Weighted $trans$	0.05	-0.676 (0.068)	-0.596 (0.12)	0.552 (0.089)	0.023 (0.008)	-0.057 (0.018)	0.082 (0.093)
	0.1	-0.683 (0.036)	-0.678 (0.065)	0.508 (0.09)	0.029 (0.006)	0.027 (0.007)	-0.227 (0.075)
	0.15	-0.653 (0.024)	-0.686 (0.042)	0.456 (0.091)	0.035 (0.007)	0.033 (0.006)	-0.382 (0.05)
	0.2	-0.612 (0.018)	-0.668 (0.029)	0.394 (0.091)	0.044 (0.012)	0.041 (0.009)	-0.469 (0.027)
	0.25	-0.567 (0.014)	-0.638 (0.021)	0.327 (0.088)	0.058 (0.023)	0.052 (0.018)	-0.519 (0.016)

Table S 4. Average correlation of degrees between twenty-nine tissues for all, cis, and $trans$ edges. For each sparse degree measure, we calculated the correlation of the degree between each tissue and averaged across all tissue pairings. Related to Figure 4

		SNP			Gene		
	τ	QV	LFDR	BH	QV	LFDR	BH
Unweighted	0.05	0.11 (0.11)	0.149 (0.115)	0.257 (0.101)	0.369 (0.093)	0.336 (0.1)	0.295 (0.123)
	0.1	0.075 (0.102)	0.093 (0.114)	0.253 (0.097)	0.377 (0.09)	0.356 (0.093)	0.336 (0.11)
	0.15	0.07 (0.093)	0.058 (0.109)	0.251 (0.095)	0.383 (0.088)	0.361 (0.091)	0.355 (0.101)
	0.2	0.077 (0.084)	0.04 (0.102)	0.255 (0.094)	0.388 (0.085)	0.364 (0.089)	0.363 (0.096)
	0.25	0.089 (0.076)	0.032 (0.094)	0.265 (0.094)	0.393 (0.082)	0.367 (0.086)	0.368 (0.093)
Weighted	0.05	0.196 (0.116)	0.261 (0.121)	0.375 (0.107)	0.369 (0.096)	0.334 (0.101)	0.306 (0.125)
	0.1	0.14 (0.108)	0.19 (0.12)	0.367 (0.1)	0.373 (0.093)	0.353 (0.094)	0.348 (0.113)
	0.15	0.119 (0.1)	0.141 (0.116)	0.36 (0.096)	0.376 (0.091)	0.357 (0.092)	0.368 (0.105)
	0.2	0.114 (0.091)	0.111 (0.11)	0.355 (0.093)	0.378 (0.088)	0.358 (0.09)	0.378 (0.1)
	0.25	0.116 (0.083)	0.093 (0.102)	0.356 (0.092)	0.379 (0.085)	0.36 (0.087)	0.385 (0.098)
Unweighted	0.05	0.175 (0.104)	0.208 (0.107)	0.252 (0.101)	0.36 (0.1)	0.285 (0.12)	0.289 (0.123)
cis	0.1	0.147 (0.1)	0.182 (0.105)	0.249 (0.097)	0.377 (0.093)	0.342 (0.106)	0.33 (0.11)
	0.15	0.134 (0.096)	0.16 (0.103)	0.248 (0.095)	0.383 (0.091)	0.364 (0.099)	0.347 (0.101)
	0.2	0.129 (0.092)	0.142 (0.101)	0.253 (0.094)	0.386 (0.089)	0.371 (0.095)	0.354 (0.095)
	0.25	0.13 (0.086)	0.13 (0.097)	0.263 (0.095)	0.388 (0.088)	0.373 (0.093)	0.356 (0.091)
Weighted	0.05	0.318 (0.108)	0.358 (0.112)	0.372 (0.107)	0.371 (0.104)	0.293 (0.122)	0.301 (0.125)
cis	0.1	0.289 (0.104)	0.345 (0.108)	0.366 (0.1)	0.39 (0.098)	0.349 (0.109)	0.342 (0.113)
	0.15	0.272 (0.101)	0.329 (0.105)	0.36 (0.096)	0.396 (0.096)	0.373 (0.102)	0.361 (0.105)
	0.2	0.264 (0.098)	0.315 (0.103)	0.357 (0.094)	0.399 (0.095)	0.381 (0.099)	0.369 (0.099)
	0.25	0.263 (0.094)	0.305 (0.1)	0.359 (0.093)	0.402 (0.094)	0.384 (0.097)	0.374 (0.097)
Unweighted	0.05	-0.714 (0.065)	-0.65 (0.104)	0.03 (0.181)	-0.001 (0.017)	-0.105 (0.022)	-0.267 (0.082)
$trans$	0.1	-0.711 (0.036)	-0.729 (0.061)	-0.019 (0.17)	0.02 (0.015)	0.006 (0.016)	-0.433 (0.058)
	0.15	-0.67 (0.025)	-0.73 (0.039)	-0.073 (0.159)	0.031 (0.016)	0.025 (0.015)	-0.49 (0.053)
	0.2	-0.621 (0.019)	-0.703 (0.027)	-0.13 (0.148)	0.044 (0.018)	0.038 (0.017)	-0.501 (0.058)
	0.25	-0.57 (0.015)	-0.663 (0.02)	-0.187 (0.136)	0.062 (0.025)	0.053 (0.022)	-0.49 (0.069)
Weighted	0.05	-0.566 (0.077)	-0.429 (0.121)	0.32 (0.197)	-0.001 (0.017)	-0.104 (0.022)	-0.241 (0.083)
$trans$	0.1	-0.617 (0.045)	-0.573 (0.074)	0.287 (0.187)	0.02 (0.015)	0.006 (0.017)	-0.411 (0.058)
	0.15	-0.605 (0.031)	-0.612 (0.051)	0.244 (0.177)	0.03 (0.016)	0.025 (0.015)	-0.469 (0.052)
	0.2	-0.575 (0.024)	-0.613 (0.037)	0.191 (0.164)	0.042 (0.018)	0.036 (0.017)	-0.483 (0.057)
	0.25	-0.537 (0.019)	-0.595 (0.028)	0.135 (0.151)	0.059 (0.023)	0.051 (0.021)	-0.474 (0.067)

Table S 5. Correlation of gene-level degree with degree of PANDA and WGCNA networks by tissue. For each tissue, PANDA and WGCNA networks were construction and the gene-level degree was calculated. We then assessed the tissue-specific correlation of the PANDA and WGCNA gene-level degree with the proposed Benjamini-Hochberg (BH) degree, thresholded with $\tau = 0.05$ and weighted by the Z-statistic. Tissue-specific sample sizes (n) are provided. Related to STAR methods

Tissue	PANDA (n)	PANDA	WGCNA (n)	WGCNA
Adipose - subcutaneous	21078	0.075	21078	-0.004
Adipose - visceral	21068	0.049	21068	-0.018
Adrenal gland	19814	0.036	19814	-0.065
Artery - aorta	20386	0.087	20386	-0.045
Artery - coronary	19838	0.009	19838	-0.085
Artery - tibial	20141	0.135	20141	-0.014
Brain - cerebellum	20768	0.084	20768	-0.057
Brain - cortex	20647	0.020	20647	-0.012
Brain - nucleus accumbens basal ganglia	20234	-0.013	20234	-0.041
Cells - cultured fibroblasts	18957	0.121	18957	0.024
Colon - sigmoid	20709	0.031	20709	-0.070
Colon - transverse	21397	0.027	21397	-0.051
Esophagus - gastroesophageal junction	20453	0.024	20453	-0.031
Esophagus - mucosa	20643	0.082	20643	-0.036
Esophagus - muscularis	20551	0.049	20551	-0.020
Heart - atrial appendage	19894	0.036	19894	0.014
Heart - left ventricle	18554	0.031	18554	0.025
Liver	18147	-0.031	18147	-0.037
Lung	21948	0.039	21948	-0.026
Muscle Skeletal	18586	0.090	18586	0.015
Nerve - tibial	21626	0.109	21626	0.051
Pancreas	19421	0.004	19421	-0.068
Pituitary	21704	0.004	21704	-0.089
Skin - not sun exposed	21372	0.092	21372	-0.044
Skin - sun exposed	21475	0.097	21475	-0.034
Spleen	20901	0.026	20901	-0.065
Stomach	20618	0.002	20618	-0.054
Thyroid	21850	0.079	21850	0.0001
Whole blood	17731	0.059	17731	0.015

Table S 6. Meta-analysis correlation of gene-level degree with degree of PANDA or WGCNA network. For each of twenty-nine tissues, we estimated the correlation between the PANDA or WGCNA degree and the weighted BH degree across all threshold τ and then meta-analyzed. Related to STAR methods

	τ	Q-value	LFDR	BH
Unweighted PANDA	0.05	0.08 (0.07, 0.1)	0.07 (0.06, 0.08)	0.05 (0.04, 0.07)
	0.1	0.09 (0.08, 0.1)	0.08 (0.07, 0.09)	0.06 (0.04, 0.07)
	0.15	0.1 (0.09, 0.1)	0.08 (0.07, 0.09)	0.06 (0.05, 0.08)
	0.2	0.1 (0.09, 0.11)	0.09 (0.08, 0.1)	0.06 (0.05, 0.08)
	0.25	0.1 (0.09, 0.1)	0.09 (0.08, 0.1)	0.07 (0.05, 0.08)
Weighted PANDA	0.05	0.08 (0.07, 0.09)	0.06 (0.05, 0.07)	0.05 (0.04, 0.07)
	0.1	0.08 (0.07, 0.09)	0.07 (0.06, 0.08)	0.06 (0.04, 0.07)
	0.15	0.09 (0.08, 0.1)	0.07 (0.06, 0.08)	0.06 (0.04, 0.07)
	0.2	0.09 (0.08, 0.1)	0.08 (0.07, 0.09)	0.06 (0.05, 0.07)
	0.25	0.1 (0.09, 0.1)	0.08 (0.07, 0.09)	0.06 (0.05, 0.08)
Unweighted WGCNA	0.05	-0.02 (-0.03, -0.01)	-0.02 (-0.04, -0.01)	-0.02 (-0.04, -0.01)
	0.1	-0.02 (-0.03, -0.01)	-0.02 (-0.03, -0.01)	-0.02 (-0.04, -0.01)
	0.15	-0.02 (-0.03, -0.01)	-0.02 (-0.03, -0.01)	-0.02 (-0.03, -0.01)
	0.2	-0.01 (-0.02, 0)	-0.01 (-0.02, 0)	-0.02 (-0.03, -0.01)
	0.25	0 (-0.01, 0)	0 (-0.01, 0.01)	-0.02 (-0.03, -0.01)
Weighted WGCNA	0.05	-0.03 (-0.04, -0.01)	-0.03 (-0.04, -0.02)	-0.03 (-0.04, -0.02)
	0.1	-0.02 (-0.03, -0.01)	-0.02 (-0.03, -0.01)	-0.03 (-0.04, -0.01)
	0.15	-0.02 (-0.03, -0.01)	-0.02 (-0.03, -0.01)	-0.03 (-0.04, -0.01)
	0.2	-0.01 (-0.02, 0)	-0.01 (-0.02, 0)	-0.03 (-0.04, -0.01)
	0.25	0 (-0.01, 0.01)	0 (-0.01, 0.01)	-0.03 (-0.04, -0.01)

Table S 7. Correlation of gene-level degree with nucleotide diversity and Tajima's D by tissue. For each tissue, we assessed the tissue-specific correlation of gene-level nucleotide diveristy and Tajima's D statistic with the proposed Benjamini-Hochberg (BH) degree, thresholded with $\tau = 0.05$ and weighted by the Z-statistic. Tissue-specific sample sizes (n) are provided. Related to STAR methods

Tissue	Nucleotide diversity (n)	Nucleotide diversity	Tajima's D (n)	Tajima's D
Adipose - subcutaneous	19043	0.158	19043	0.157
Adipose - visceral	17561	0.136	17561	0.141
Adrenal gland	13373	0.106	13373	0.122
Artery - aorta	17159	0.160	17159	0.160
Artery - coronary	11762	0.083	11762	0.102
Artery - tibial	18258	0.179	18258	0.172
Brain - cerebellum	16368	0.132	16368	0.143
Brain - cortex	14277	0.100	14277	0.124
Brain - nucleus accumbens	12999	0.077	12999	0.111
Cells - cultured fibroblasts	17360	0.215	17360	0.195
Colon - sigmoid	15804	0.112	15804	0.126
Colon - transverse	16883	0.105	16883	0.124
Esophagus - gastroesophageal junction	15983	0.114	15983	0.124
Esophagus - mucosa	18310	0.179	18310	0.177
Esophagus - muscularis	18060	0.156	18060	0.160
Heart - atrial appendage	15928	0.141	15928	0.146
Heart - left ventricle	14330	0.134	14330	0.149
Liver	10403	0.088	10403	0.108
Lung	18963	0.143	18963	0.146
Muscle Skeletal	16532	0.160	16532	0.173
Nerve - tibial	20323	0.199	20323	0.188
Pancreas	14885	0.133	14885	0.143
Pituitary	15258	0.081	15258	0.098
Skin - not sun exposed	19077	0.157	19077	0.162
Skin - sun exposed	19755	0.162	19755	0.165
Spleen	15766	0.125	15766	0.139
Stomach	14539	0.084	14539	0.108
Thyroid	20519	0.175	20519	0.171
Whole blood	15743	0.169	15743	0.161

Table S 8. Meta-analysis correlation of gene-level degree with nucleotide diversity and Tajima's D. For each of twenty-nine tissues, we estimated the correlation between nucleotide diversity or Tajima's D and the weighted BH degree across all threshold τ and then meta-analyzed. Related to STAR methods

	τ	Q-value	LFDR	BH
Unweighted nucleotide diversity	0.05	0.19 (0.18, 0.2)	0.16 (0.15, 0.17)	0.14 (0.13, 0.16)
	0.1	0.19 (0.18, 0.2)	0.18 (0.17, 0.19)	0.16 (0.15, 0.18)
	0.15	0.19 (0.19, 0.2)	0.18 (0.17, 0.19)	0.17 (0.16, 0.19)
	0.2	0.2 (0.19, 0.2)	0.18 (0.18, 0.19)	0.18 (0.16, 0.19)
	0.25	0.19 (0.19, 0.2)	0.19 (0.18, 0.19)	0.18 (0.17, 0.2)
Weighted nucleotide diversity	0.05	0.18 (0.17, 0.19)	0.15 (0.14, 0.16)	0.14 (0.12, 0.15)
	0.1	0.18 (0.17, 0.19)	0.17 (0.16, 0.18)	0.16 (0.14, 0.17)
	0.15	0.18 (0.18, 0.19)	0.17 (0.16, 0.18)	0.17 (0.15, 0.18)
	0.2	0.19 (0.18, 0.2)	0.17 (0.16, 0.18)	0.17 (0.16, 0.19)
	0.25	0.19 (0.18, 0.2)	0.17 (0.17, 0.18)	0.18 (0.16, 0.19)
Unweighted Tajima's D	0.05	0.17 (0.16, 0.18)	0.15 (0.14, 0.16)	0.15 (0.14, 0.16)
	0.1	0.17 (0.16, 0.17)	0.16 (0.15, 0.17)	0.16 (0.15, 0.17)
	0.15	0.17 (0.16, 0.17)	0.16 (0.16, 0.17)	0.16 (0.15, 0.17)
	0.2	0.16 (0.16, 0.17)	0.16 (0.16, 0.17)	0.17 (0.16, 0.18)
	0.25	0.16 (0.16, 0.17)	0.16 (0.16, 0.17)	0.17 (0.16, 0.18)
Weighted Tajima's D	0.05	0.16 (0.16, 0.17)	0.15 (0.14, 0.16)	0.14 (0.13, 0.15)
	0.1	0.17 (0.16, 0.17)	0.16 (0.15, 0.17)	0.16 (0.15, 0.17)
	0.15	0.17 (0.16, 0.17)	0.16 (0.15, 0.17)	0.16 (0.15, 0.17)
	0.2	0.17 (0.16, 0.17)	0.16 (0.15, 0.17)	0.17 (0.16, 0.18)
	0.25	0.17 (0.16, 0.17)	0.16 (0.15, 0.17)	0.17 (0.16, 0.18)

Table S 9. Heritability analysis between SNP-level and gene-level degree and blood-related traits. We performed LD score regression to assess the enrichment of the SNP-level and gene-level Benjamini-Hochberg (BH) degree, thresholded with $\tau = 0.05$ and weighted by the Z-statistic, in blood-related traits and relevant tissues. Related to Figure 5

Tissue	Trait	SNP-level Enrichment (P)	SNP-level Coefficient (SE)	Gene-level Enrichment (P)	Gene-level Coefficient (SE)
Artery - aorta	Eosinophil	2.05 (5.92E-03)	7.64E-10 (1.64E-08)	1.32 (6.09E-03)	-6.35E-09 (5.63E-09)
Artery - aorta	Platelet	2.42 (6.62E-04)	3.40E-09 (2.89E-08)	1.56 (1.09E-06)	-5.77E-09 (9.24E-09)
Artery - aorta	RBC Width	3.29 (8.84E-06)	1.95E-08 (2.20E-08)	1.91 (1.55E-09)	4.97E-09 (7.16E-09)
Artery - aorta	Red Cell Count	2.47 (9.92E-06)	6.22E-09 (1.36E-08)	1.53 (2.95E-06)	-4.06E-10 (5.45E-09)
Artery - aorta	White Cell Count	2.02 (1.06E-04)	-2.18E-09 (1.11E-08)	1.34 (1.99E-05)	-5.68E-09 (3.71E-09)
Artery - aorta	HDL	2.09 (3.42E-03)	-2.08E-09 (8.85E-09)	1.26 (4.16E-02)	-7.04E-09 (3.90E-09)
Artery - aorta	LDL	2.09 (1.90E-02)	-9.84E-09 (9.64E-09)	1.72 (2.08E-04)	1.53E-09 (4.51E-09)
Artery - cor.	Eosinophil	1.53 (2.28E-01)	-1.53E-08 (1.88E-08)	1.37 (1.19E-02)	-4.54E-09 (6.60E-09)
Artery - cor.	Platelet	2.01 (8.59E-02)	-1.12E-08 (3.77E-08)	1.54 (1.75E-04)	-9.23E-09 (9.45E-09)
Artery - cor.	RBC Width	3.67 (4.25E-03)	3.79E-08 (3.69E-08)	2.25 (3.49E-08)	1.53E-08 (9.41E-09)
Artery - cor.	Red Cell Count	2.00 (7.65E-02)	-9.96E-09 (2.40E-08)	1.44 (1.27E-03)	-7.60E-09 (6.04E-09)
Artery - cor.	White Cell Count	1.73 (6.94E-02)	-6.78E-09 (1.65E-08)	1.32 (7.63E-04)	-6.65E-09 (4.09E-09)
Artery - cor.	HDL	1.57 (1.71E-01)	-1.26E-08 (9.90E-09)	1.27 (6.26E-02)	-6.83E-09 (3.90E-09)
Artery - cor.	LDL	1.47 (5.01E-01)	-1.84E-08 (1.33E-08)	1.84 (5.17E-03)	2.36E-09 (6.53E-09)
Artery - tibial	Eosinophil	2.21 (2.15E-04)	8.00E-09 (1.44E-08)	1.32 (2.21E-03)	-7.15E-09 (5.27E-09)
Artery - tibial	Platelet	2.67 (1.93E-06)	2.10E-08 (2.42E-08)	1.54 (9.91E-07)	-9.29E-09 (8.82E-09)
Artery - tibial	RBC Width	3.29 (1.11E-07)	2.12E-08 (1.87E-08)	1.88 (7.71E-08)	2.52E-09 (7.87E-09)
Artery - tibial	Red Cell Count	2.50 (4.55E-08)	8.53E-09 (1.14E-08)	1.31 (9.50E-04)	-1.57E-08 (4.50E-09)
Artery - tibial	White Cell Count	2.15 (1.77E-07)	3.74E-09 (9.58E-09)	1.31 (1.22E-05)	-7.75E-09 (3.59E-09)
Artery - tibial	HDL	2.03 (4.10E-04)	-4.14E-09 (7.30E-09)	1.31 (3.89E-03)	-4.94E-09 (3.23E-09)
Artery - tibial	LDL	2.17 (3.05E-03)	-9.20E-09 (9.06E-09)	1.57 (3.84E-03)	-2.44E-09 (5.06E-09)
Heart atr. app.	Eosinophil	2.19 (3.90E-03)	7.01E-09 (1.76E-08)	1.43 (3.37E-04)	-8.76E-10 (5.66E-09)
Heart atr. app.	Platelet	2.38 (2.18E-03)	-8.34E-11 (3.04E-08)	1.61 (6.74E-08)	-2.11E-09 (7.91E-09)
Heart atr. app.	RBC Width	3.73 (2.55E-06)	3.72E-08 (2.34E-08)	1.99 (7.72E-09)	6.58E-09 (7.55E-09)
Heart atr. app.	Red Cell Count	2.84 (7.35E-06)	2.55E-08 (1.74E-08)	1.43 (1.61E-04)	-7.08E-09 (5.71E-09)
Heart atr. app.	White Cell Count	2.21 (5.02E-04)	6.86E-09 (1.47E-08)	1.33 (3.67E-05)	-6.26E-09 (3.81E-09)
Heart atr. app.	HDL	2.26 (5.12E-03)	1.89E-09 (1.03E-08)	1.50 (4.61E-04)	9.00E-10 (4.08E-09)
Heart atr. app.	LDL	2.12 (2.56E-02)	-9.63E-09 (9.60E-09)	2.14 (5.74E-06)	1.16E-08 (5.17E-09)
Heart left vent.	Eosinophil	2.44 (2.33E-03)	1.74E-08 (2.02E-08)	1.49 (4.44E-05)	-1.97E-09 (5.50E-09)
Heart left vent.	Platelet	2.35 (3.03E-03)	-4.24E-09 (3.05E-08)	1.72 (5.26E-09)	-7.29E-10 (8.52E-09)
Heart left vent.	RBC Width	3.92 (1.25E-05)	4.25E-08 (2.73E-08)	2.12 (1.18E-08)	7.69E-09 (8.50E-09)
Heart left vent.	Red Cell Count	2.84 (2.51E-05)	2.26E-08 (1.88E-08)	1.48 (8.58E-05)	-1.01E-08 (6.04E-09)
Heart left vent.	White Cell Count	2.26 (7.55E-04)	7.91E-09 (1.61E-08)	1.51 (6.92E-08)	-5.96E-10 (4.34E-09)
Heart left vent.	HDL	2.14 (7.31E-03)	-2.54E-09 (1.02E-08)	1.48 (2.67E-03)	-2.61E-09 (4.42E-09)
Heart left vent.	LDL	2.34 (1.92E-02)	-5.86E-09 (1.10E-08)	2.23 (2.06E-06)	1.11E-08 (5.21E-09)
Whole blood	Eosinophil	3.04 (1.50E-05)	2.62E-08 (1.98E-08)	1.86 (3.75E-08)	1.70E-09 (7.36E-09)
Whole blood	Platelet	3.19 (1.84E-07)	4.28E-08 (2.74E-08)	1.99 (2.33E-10)	6.89E-09 (1.16E-08)
Whole blood	RBC Width	4.25 (2.06E-09)	5.20E-08 (2.13E-08)	2.45 (2.25E-11)	1.58E-08 (9.03E-09)
Whole blood	Red Cell Count	3.03 (1.01E-06)	2.11E-08 (1.93E-08)	1.71 (3.87E-08)	-5.90E-09 (6.80E-09)
Whole blood	White Cell Count	2.83 (1.36E-08)	2.07E-08 (1.35E-08)	1.74 (2.41E-11)	5.22E-10 (5.15E-09)
Whole blood	HDL	2.58 (1.49E-05)	3.20E-09 (8.51E-09)	1.65 (3.06E-03)	-2.52E-09 (7.15E-09)
Whole blood	LDL	2.38 (4.34E-03)	-9.41E-09 (1.05E-08)	1.81 (6.48E-04)	-3.97E-09 (5.36E-09)

Table S 10. Heritability analysis between SNP-level and gene-level degree and blood-related traits. We performed LD score regression to assess the enrichment of the SNP-level and gene-level Q-value (QV) degree, thresholded with $\tau = 0.05$ and weighted by the Z-statistic, in blood-related traits and relevant tissues. Related to Figure 5

Tissue	Trait	SNP-level Enrichment (P)	SNP-level Coefficient (SE)	Gene-level Enrichment (P)	Gene-level Coefficient (SE)
Artery - aorta	Eosinophil	2.05 (1.59E-03)	3.31E-09 (1.46E-08)	1.30 (3.05E-03)	-6.67E-09 (5.22E-09)
Artery - aorta	Platelet	2.35 (3.49E-05)	3.61E-09 (2.35E-08)	1.57 (1.93E-09)	-1.20E-09 (8.12E-09)
Artery - aorta	RBC Width	3.04 (8.32E-07)	1.55E-08 (1.81E-08)	1.79 (1.84E-11)	2.61E-09 5.40E-09)
Artery - aorta	Red Cell Count	2.43 (6.79E-08)	9.59E-09 (1.11E-08)	1.45 (4.77E-07)	-3.86E-09 (4.51E-09)
Artery - aorta	White Cell Count	2.01 (3.34E-06)	1.32E-10 (9.42E-09)	1.33 (1.31E-06)	-5.29E-09 (3.35E-09)
Artery - aorta	HDL	1.96 (1.87E-03)	-3.70E-09 (7.86E-09)	1.24 (2.45E-02)	-6.56E-09 (3.61E-09)
Artery - aorta	LDL	2.26 (5.99E-03)	-4.00E-09 (1.00E-08)	1.60 (2.28E-04)	-2.22E-11 (3.90E-09)
Artery - cor.	Eosinophil	1.34 (3.40E-01)	-1.99E-08 (1.60E-08)	1.32 (3.01E-04)	-3.63E-09 (4.32E-09)
Artery - cor.	Platelet	1.84 (3.89E-02)	-1.25E-08 (2.74E-08)	1.43 (1.39E-07)	-1.08E-08 (6.45E-09)
Artery - cor.	RBC Width	2.77 (1.82E-03)	1.51E-08 (2.36E-08)	1.86 (1.50E-10)	7.80E-09 (5.63E-09)
Artery - cor.	Red Cell Count	1.87 (1.09E-02)	-7.34E-09 (1.49E-08)	1.37 (5.34E-05)	-8.36E-09 (4.48E-09)
Artery - cor.	White Cell Count	1.63 (3.00E-02)	-7.07E-09 (1.24E-08)	1.36 (1.45E-08)	-2.39E-09 (3.16E-09)
Artery - cor.	HDL	1.46 (1.43E-01)	-1.03E-08 (8.37E-09)	1.38 (4.72E-03)	-1.48E-09 (4.12E-09)
Artery - cor.	LDL	1.52 (3.13E-01)	-1.33E-08 (1.04E-08)	1.62 (5.33E-04)	9.74E-10 (4.42E-09)
Artery - tibial	Eosinophil	2.08 (2.78E-04)	3.87E-09 (1.35E-08)	1.26 (5.54E-03)	-9.86E-09 (4.82E-09)
Artery - tibial	Platelet	2.56 (6.00E-08)	1.86E-08 (2.05E-08)	1.52 (4.01E-07)	-7.87E-09 (8.79E-09)
Artery - tibial	RBC Width	3.08 (3.81E-08)	1.74E-08 (1.71E-08)	1.78 (2.87E-08)	5.67E-10 (6.95E-09)
Artery - tibial	Red Cell Count	2.36 (8.69E-09)	5.52E-09 (1.02E-08)	1.30 (4.07E-04)	-1.40E-08 (4.45E-09)
Artery - tibial	White Cell Count	2.12 (1.03E-08)	4.38E-09 (8.80E-09)	1.32 (4.47E-06)	-6.25E-09 (3.59E-09)
Artery - tibial	HDL	1.95 (1.87E-04)	-4.90E-09 (6.52E-09)	1.27 (7.43E-03)	-5.26E-09 (3.23E-09)
Artery - tibial	LDL	2.55 (3.81E-04)	2.36E-09 (9.46E-09)	1.51 (2.14E-03)	-2.69E-09 (4.29E-09)
Heart atr. app.	Eosinophil	2.12 (1.09E-03)	6.83E-09 (1.49E-08)	1.41 (1.02E-04)	2.97E-10 (5.00E-09)
Heart atr. app.	Platelet	2.42 (3.98E-05)	9.41E-09 (2.42E-08)	1.58 (5.39E-12)	1.09E-09 (5.70E-09)
Heart atr. app.	RBC Width	3.36 (1.66E-07)	2.99E-08 (1.88E-08)	1.82 (1.58E-09)	4.50E-09 (6.40E-09)
Heart atr. app.	Red Cell Count	2.57 (3.38E-07)	1.76E-08 (1.37E-08)	1.38 (2.69E-05)	-8.06E-09 (4.73E-09)
Heart atr. app.	White Cell Count	2.04 (8.84E-05)	1.76E-09 (1.16E-08)	1.34 (7.42E-07)	-4.30E-09 (3.29E-09)
Heart atr. app.	HDL	2.13 (7.74E-04)	1.33E-09 (7.87E-09)	1.37 (1.72E-03)	-1.37E-09 (3.51E-09)
Heart atr. app.	LDL	1.92 (2.22E-02)	-1.23E-08 (8.29E-09)	1.76 (1.76E-05)	4.84E-09 (3.87E-09)
Heart left vent.	Eosinophil	2.24 (8.04E-04)	1.12E-08 (1.64E-08)	1.43 (1.61E-05)	-3.07E-09 (4.72E-09)
Heart left vent.	Platelet	2.42 (3.01E-05)	6.28E-09 (2.38E-08)	1.70 (3.60E-10)	4.12E-09 (8.27E-09)
Heart left vent.	RBC Width	3.40 (8.94E-07)	2.91E-08 (2.09E-08)	1.96 (1.85E-11)	6.31E-09 (6.37E-09)
Heart left vent.	Red Cell Count	2.69 (1.93E-07)	2.10E-08 (1.43E-08)	1.53 (1.68E-07)	-3.40E-09 (5.07E-09)
Heart left vent.	White Cell Count	2.30 (1.43E-05)	1.24E-08 (1.34E-08)	1.44 (6.98E-09)	-2.51E-09 (3.65E-09)
Heart left vent.	HDL	2.14 (5.18E-04)	1.77E-10 (7.73E-09)	1.57 (2.95E-04)	2.11E-09 (5.28E-09)
Heart left vent.	LDL	2.21 (5.74E-03)	-6.69E-09 (8.59E-09)	1.98 (6.61E-07)	7.80E-09 (3.95E-09)
Whole blood	Eosinophil	2.80 (1.18E-05)	1.96E-08 (1.78E-08)	1.85 (1.39E-09)	2.96E-09 (6.52E-09)
Whole blood	Platelet	2.90 (1.03E-08)	2.98E-08 (2.24E-08)	1.93 (3.04E-12)	5.16E-09 (9.83E-09)
Whole blood	RBC Width	3.92 (1.58E-10)	4.63E-08 (1.88E-08)	2.26 (2.66E-12)	9.29E-09 (7.95E-09)
Whole blood	Red Cell Count	2.94 (9.40E-09)	2.49E-08 (1.62E-08)	1.63 (6.87E-09)	-9.90E-09 (5.86E-09)
Whole blood	White Cell Count	2.70 (1.22E-10)	1.95E-08 (1.12E-08)	1.70 (6.58E-13)	-8.12E-10 (4.66E-09)
Whole blood	HDL	2.55 (1.90E-06)	6.10E-09 (8.25E-09)	1.59 (1.84E-03)	-3.89E-09 (6.58E-09)
Whole blood	LDL	2.70 (3.31E-04)	1.38E-09 (1.01E-08)	1.86 (6.41E-05)	-1.20E-09 (5.12E-09)

Table S 11. Heritability analysis between SNP-level and gene-level degree and blood-related traits. We performed LD score regression to assess the enrichment of the SNP-level and gene-level LFDR degree, thresholded with $\tau = 0.05$ and weighted by the Z-statistic, in blood-related traits and relevant tissues. Related to Figure 5

Tissue	Trait	SNP-level Enrichment (P)	SNP-level Coefficient (SE)	Gene-level Enrichment (P)	Gene-level Coefficient (SE)
Artery - aorta	Eosinophil	2.12 (2.76E-03)	5.88E-09 (1.59E-08)	1.34 (1.07E-03)	-6.63E-09 (5.28E-09)
Artery - aorta	Platelet	2.40 (5.96E-04)	4.81E-09 (2.83E-08)	1.65 (3.66E-10)	2.36E-09 (8.50E-09)
Artery - aorta	RBC Width	3.20 (1.24E-05)	1.78E-08 (2.15E-08)	1.84 (4.30E-11)	2.26E-09 (5.92E-09)
Artery - aorta	Red Cell Count	2.38 (1.74E-05)	3.88E-09 (1.33E-08)	1.47 (6.68E-07)	-4.99E-09 (4.66E-09)
Artery - aorta	White Cell Count	2.04 (4.03E-05)	3.89E-10 (1.07E-08)	1.37 (1.61E-07)	-4.82E-09 (3.48E-09)
Artery - aorta	HDL	2.01 (3.35E-03)	-3.53E-09 (8.26E-09)	1.24 (3.73E-02)	-7.65E-09 (3.90E-09)
Artery - aorta	LDL	2.06 (1.81E-02)	-9.75E-09 (9.38E-09)	1.62 (5.24E-04)	-7.66E-10 (4.10E-09)
Artery - cor.	Eosinophil	1.43 (3.14E-01)	-1.72E-08 (1.85E-08)	1.33 (1.45E-03)	-5.54E-09 (5.05E-09)
Artery - cor.	Platelet	1.86 (8.26E-02)	-1.53E-08 (3.27E-08)	1.52 (2.73E-08)	-6.83E-09 (6.94E-09)
Artery - cor.	RBC Width	3.06 (5.35E-03)	2.00E-08 (2.96E-08)	1.93 (2.39E-12)	6.88E-09 (5.39E-09)
Artery - cor.	Red Cell Count	1.90 (5.04E-02)	-1.04E-08 (1.96E-08)	1.43 (6.95E-06)	-7.14E-09 (4.67E-09)
Artery - cor.	White Cell Count	1.69 (5.66E-02)	-6.01E-09 (1.50E-08)	1.38 (1.38E-08)	-3.27E-09 (3.44E-09)
Artery - cor.	HDL	1.36 (3.29E-01)	-1.52E-08 (9.16E-09)	1.35 (2.42E-02)	-3.74E-09 (4.84E-09)
Artery - cor.	LDL	1.23 (7.00E-01)	-2.13E-08 (1.19E-08)	1.61 (1.83E-03)	-1.27E-09 (4.71E-09)
Artery - tibial	Eosinophil	2.22 (2.44E-04)	9.97E-09 (1.44E-08)	1.29 (2.28E-03)	-9.72E-09 (4.91E-09)
Artery - tibial	Platelet	2.59 (6.73E-06)	1.78E-08 (2.45E-08)	1.59 (1.17E-08)	-4.73E-09 (8.27E-09)
Artery - tibial	RBC Width	3.28 (1.92E-07)	2.25E-08 (1.88E-08)	1.85 (7.16E-09)	1.21E-09 (7.27E-09)
Artery - tibial	Red Cell Count	2.46 (1.31E-07)	8.35E-09 (1.14E-08)	1.31 (2.52E-04)	-1.60E-08 (4.22E-09)
Artery - tibial	White Cell Count	2.11 (7.01E-07)	2.75E-09 (9.62E-09)	1.32 (2.74E-06)	-7.67E-09 (3.63E-09)
Artery - tibial	HDL	1.97 (7.74E-04)	-4.89E-09 (7.35E-09)	1.31 (1.56E-03)	-5.04E-09 (3.15E-09)
Artery - tibial	LDL	2.10 (5.09E-03)	-9.77E-09 (9.07E-09)	1.54 (3.30E-03)	-3.26E-09 (4.88E-09)
Heart atr. app.	Eosinophil	2.20 (3.75E-03)	9.31E-09 (1.78E-08)	1.37 (3.03E-04)	-5.01E-09 (4.86E-09)
Heart atr. app.	Platelet	2.29 (2.26E-03)	-3.10E-09 (2.90E-08)	1.59 (1.73E-11)	-3.03E-09 (5.86E-09)
Heart atr. app.	RBC Width	3.60 (4.27E-06)	3.50E-08 (2.29E-08)	1.92 (7.51E-10)	5.03E-09 (6.78E-09)
Heart atr. app.	Red Cell Count	2.74 (7.15E-06)	2.29E-08 (1.65E-08)	1.40 (2.64E-05)	-9.96E-09 (4.94E-09)
Heart atr. app.	White Cell Count	2.17 (3.94E-04)	6.90E-09 (1.41E-08)	1.37 (8.75E-08)	-5.40E-09 (3.35E-09)
Heart atr. app.	HDL	2.22 (3.64E-03)	2.27E-09 (9.62E-09)	1.45 (5.64E-04)	-7.04E-10 (3.74E-09)
Heart atr. app.	LDL	1.98 (3.63E-02)	-1.16E-08 (9.13E-09)	1.93 (5.65E-05)	7.07E-09 (5.01E-09)
Heart left vent.	Eosinophil	2.45 (1.35E-03)	1.94E-08 (1.93E-08)	1.45 (1.17E-05)	-3.97E-09 (4.75E-09)
Heart left vent.	Platelet	2.45 (7.26E-04)	5.14E-09 (2.89E-08)	1.67 (3.87E-11)	-2.51E-09 (7.29E-09)
Heart left vent.	RBC Width	3.73 (1.18E-05)	3.75E-08 (2.57E-08)	2.07 (1.15E-10)	8.81E-09 (7.55E-09)
Heart left vent.	Red Cell Count	2.79 (1.44E-05)	2.20E-08 (1.78E-08)	1.47 (1.02E-06)	-9.67E-09 (4.95E-09)
Heart left vent.	White Cell Count	2.31 (2.45E-04)	1.14E-08 (1.55E-08)	1.48 (2.67E-09)	-2.36E-09 (3.87E-09)
Heart left vent.	HDL	2.14 (4.00E-03)	-1.32E-09 (9.31E-09)	1.62 (1.87E-04)	2.15E-09 (5.34E-09)
Heart left vent.	LDL	2.28 (1.91E-02)	-6.32E-09 (1.05E-08)	2.06 (4.56E-07)	8.17E-09 (4.05E-09)
Whole blood	Eosinophil	3.02 (1.62E-05)	2.71E-08 (1.97E-08)	1.85 (1.42E-09)	2.22E-09 (6.70E-09)
Whole blood	Platelet	3.13 (4.14E-07)	4.15E-08 (2.75E-08)	2.01 (4.76E-12)	1.10E-08 (1.09E-08)
Whole blood	RBC Width	4.24 (2.21E-09)	5.39E-08 (2.12E-08)	2.40 (1.49E-13)	1.53E-08 (8.04E-09)
Whole blood	Red Cell Count	2.99 (1.28E-06)	2.13E-08 (1.92E-08)	1.69 (2.81E-09)	-6.81E-09 (6.22E-09)
Whole blood	White Cell Count	2.85 (1.28E-08)	2.29E-08 (1.35E-08)	1.73 (1.04E-13)	2.62E-10 (4.74E-09)
Whole blood	HDL	2.60 (1.61E-05)	4.89E-09 (8.68E-09)	1.66 (8.93E-04)	-2.23E-09 (6.63E-09)
Whole blood	LDL	2.37 (3.78E-03)	-8.55E-09 (1.03E-08)	1.92 (1.72E-05)	-6.61E-10 (4.86E-09)

Table S 12. Computational cost across network construction and degree calculation approaches given for SNPs across all thresholds τ. Related to STAR methods

Step	Approach	Average time (sd)	Output size (.Rds)
1	Calculate edges for thresholded, location-specific methods.	154.9 (12.8) s	482 KB
	Calculate edges for exhaustive, genome-wide methods.	1104.4 (80.9) s	2093 KB
2	Calculate BH-based SNP degrees.	0.2 (0.03) s	347 KB
	Calculate QV-based SNP degrees.	0.4 (0.02) s	537 KB
	Calculate LFDR-based SNP degrees.	0.3 (0.02) s	401 KB
	Calculate NP-based SNP degrees.	0.1 (0.01) s	161 KB
